# Imaging and Clinical Correlates of [^177^Lu]Lu-PSMA PET-Defined Eligibility in De Novo Metastatic Prostate Cancer

**DOI:** 10.3390/biomedicines14081818

**Published:** 2026-08-13

**Authors:** Giovanna Pecoraro, Marco Cuzzocrea, Cesare Michele Iacovitti, Marialuisa Puglisi, Chiara Martinello, Alberto De Giorgi, Sara Merler, Luigi Tortola, Hui-Ming Lin, Gianmarco Leone, Fabio Turco, Ricardo Pereira Mestre, Giorgio Treglia, Ursula Vogl, Silke Gillessen, Martino Pedrani, Gaetano Paone

**Affiliations:** 1 Oncology Institute of Southern Switzerland (IOSI), Ente Ospedaliero Cantonale (EOC), 6500 Bellinzona, Switzerland; giovanna.pecoraro@eoc.ch (G.P.); marialuisa.puglisi@eoc.ch (M.P.); alberto.degiorgi@eoc.ch (A.D.G.); luigi.tortola@eoc.ch (L.T.); huiming.lin@eoc.ch (H.-M.L.); gianmarco.leone@eoc.ch (G.L.); fabio.turco@eoc.ch (F.T.); ricardo.pereiramestre@eoc.ch (R.P.M.); ursula.vogl@eoc.ch (U.V.); silke.gillessensommer@eoc.ch (S.G.); 2 Institute of Oncology Research (IOR), 6500 Bellinzona, Switzerland; 3 Department of Clinical Medicine and Surgery, University of Naples “Federico II”, 80131 Naples, Italy; 4 Clinic of Nuclear Medicine, Institute of Integrated Diagnostics of Southern Switzerland (IDISI), Ente Ospedaliero Cantonale (EOC), 6500 Bellinzona, Switzerland; marco.cuzzocrea@eoc.ch (M.C.); cesaremichele.iacovitti@eoc.ch (C.M.I.); chiara.martinello@eoc.ch (C.M.); giorgio.treglia@eoc.ch (G.T.); gaetano.paone@eoc.ch (G.P.); 5 Medical Oncology Department, Turin University, 10124 Turin, Italy; 6 South Tyrolean Healthcare Service, 39100 Bolzano, Italy; sara.merler@sabes.it; 7 Cancer Institute, Department of Oncology, University College London, London WC1E 6DD, UK; 8 Faculty of Biomedical Sciences, Università della Svizzera Italiana, 6962 Lugano, Switzerland; 9 Faculty of Biology and Medicine, University of Lausanne, 1011 Lausanne, Switzerland

**Keywords:** PSMA PET/CT, [^177^Lu]Lu-PSMA, radioligand therapy, prostate cancer, exploratory modelling, PSMA PET-defined eligibility, treatment eligibility

## Abstract

**Objectives:** Eligibility for Lutetium-177 labeled prostate-specific membrane antigen ([^177^Lu]Lu-PSMA) radioligand therapy depends on PSMA PET/CT interpretation and may vary across observers and centres. We aimed to identify imaging and clinical correlates of VISION-like PSMA PET-defined eligibility and to compare exploratory clinical, PET-based, and combined models. **Materials and Methods:** Seventy-six consecutive patients with de novo metastatic prostate cancer undergoing PSMA PET/CT were retrospectively assessed for [^177^Lu]Lu-PSMA eligibility. Candidacy was determined by consensus of two nuclear medicine physicians using VISION-like criteria. Three stepwise logistic regression models used clinical variables, PSMA PET/CT variables, or both, and were internally validated with bootstrap out-of-bag predictions. **Results:** Forty-three patients (56.6%) met VISION-like criteria for RLT. Internally validated area under the curve (AUC) values were 0.731, 0.817, and 0.829 for the clinical, PSMA PET/CT, and combined models. PET-defined candidacy was associated with higher PSMA-positive lesion count, greater PET-derived tumour burden, and higher mean standardised uptake value (SUVmean). Clinically, CHAARTED low-volume disease and prior docetaxel exposure were linked to lower probability, whereas disease state at imaging was not significant. In the combined multivariable model, SUVmean was independently associated with higher candidacy (odds ratio [OR]: 1.22, 95% confidence interval [CI]: 1.04–1.42; *p* = 0.015), whereas prior docetaxel showed the opposite association (OR: 0.10, 95% CI: 0.0129–0.776; *p* = 0.028). **Conclusions:** PSMA-positive lesion count and SUVmean were the most reproducible determinants of VISION-like eligibility. Prior docetaxel exposure was associated with lower candidacy in the combined model, although the exposed subgroup was small and the confidence interval wide. Whether systemic therapy modifies PSMA expression, and with it access to subsequent PSMA-targeted lines, requires paired imaging before and after treatment in the same patients.

## 1. Introduction

Over the past decade, Lutetium-177 labeled prostate-specific membrane antigen ([^177^Lu]Lu-PSMA) radioligand therapy (RLT) has emerged as a transformative option for metastatic castration-resistant prostate cancer (mCRPC), selectively delivering radiation to prostate-specific membrane antigen (PSMA)-expressing tumour sites and improving survival and quality of life [1,2,3,4,5,6,7]. Phase III trials have evaluated [^177^Lu]Lu-PSMA across mCRPC [5,6,7,8,9] and, more recently, metastatic hormone-sensitive prostate cancer (mHSPC) [10,11,12]. The pivotal VISION trial established a survival benefit and supported regulatory approval, underscoring the central role of molecular imaging in patient selection [6,13].

In practice, candidacy is determined first by sufficient PSMA expression on PSMA PET/CT—typically uptake exceeding the hepatic background with no PSMA-negative measurable lesions—and then by clinical factors such as performance status, organ function, histology, and prior therapies, usually within a multidisciplinary tumour board [13,14]. However, current selection still relies largely on visual or semi-quantitative assessment, which is vulnerable to inter-observer variability and inter-centre differences in acquisition and interpretation, and may not capture the heterogeneity of PSMA expression across metastatic sites [14,15,16,17,18].

Accordingly, there is growing interest in the clinical and pathological correlates of PSMA expression. Tumour histology, disease burden, ISUP grade, visceral involvement, and biochemical markers have been associated with PSMA uptake [19,20,21], and quantitative PSMA PET metrics such as SUVmax, SUVmean, and total lesion activity carry prognostic and predictive value [22,23]. Yet the incremental value of these imaging features over standard clinical variables remains undefined, and no consensus framework exists for integrating them into eligibility assessment.

More broadly, precision diagnosis in prostate cancer increasingly integrates imaging phenotypes with molecular information. Studies of driver alterations, genomic instability, tumour-microenvironment features, and multimodal classification indicate that PSMA expression represents only one component of biological heterogeneity. The complementary value of genomic, transcriptomic, immune, and imaging markers should therefore be examined in future eligibility models, particularly when PSMA PET findings are heterogeneous [24,25,26].

We therefore performed exploratory development and internal validation of logistic regression models relating clinical and PSMA PET parameters to expert-defined, VISION-like PSMA PET eligibility. We compared clinical-only, imaging-only, and combined approaches to identify the features most consistently associated with imaging classification. These models were intended to formalise expert PET assessment and generate hypotheses; they were not designed to predict treatment benefit or replace multidisciplinary determination of clinical candidacy.

## 2. Materials and Methods

### 2.1. Study Design and Patient Selection

This retrospective, single-centre study was conducted under a retrospective data-collection protocol approved by the local ethics committee (protocol 2023-01208) and in accordance with the Declaration of Helsinki and comparable ethical standards; informed consent was handled according to the approved protocol. We included 76 consecutive men with de novo metastatic prostate cancer who underwent PSMA PET/CT in the mHSPC or mCRPC setting, together with CT and bone scintigraphy at mHSPC diagnosis, between January 2020 and December 2024. Inclusion criteria were histologically confirmed prostate adenocarcinoma and de novo metastatic disease. Exclusion criteria were previous [^177^Lu]Lu-PSMA therapy, absence of baseline PSMA PET/CT, or incomplete clinical data.

Collected clinical variables included age at diagnosis and ECOG performance status; pathological variables included ISUP grade group, neuroendocrine differentiation, and histological subtype. Radiological variables comprised visceral and bone metastases and volume of disease according to CHAARTED criteria, assigned from conventional CT and bone scintigraphy at initial mHSPC presentation. PSMA PET-derived variables were measured at the study PET time point, which occurred later for patients imaged after progression to mCRPC. This temporal distinction was considered when interpreting associations between baseline disease volume and later PET findings. PET parameters included visual assessment informed by PROMISE terminology and quantitative metrics: reference-lesion SUVmax (RL-SUVmax), SUVmean, total PSMA tumour volume (tPSMA-TV), tumour lesion activity (TLA; SUVmean × tPSMA-TV), and hepatic SUVmax [27]. Exploratory volumetric parameters included PSMA-TV thresholded at the salivary gland SUVmax (PSMA-TVsal) and the percentage of tPSMA-TV exceeding this threshold (PSMA-TV%). miTNM categories were not systematically recorded and PROMISE terminology only informed visual interpretation.

### 2.2. PET/CT Imaging Analysis

All patients underwent PSMA PET/CT on a Biograph mCT system (Siemens Healthineers, Zürich, Switzerland). Imaging was acquired 60 min after injection of 2–3 MBq/kg [^68^Ga]Ga-PSMA-11 (Locametz; Novartis, Basel, Switzerland), with low-dose CT for attenuation correction. PET data were reconstructed using three-dimensional ordered-subset expectation maximisation (4 iterations and 24 subsets; 400 × 400 matrix; Gaussian filter, with full width at half maximum of 4.0 mm) with standard attenuation, scatter, and random corrections. Quantitative SUV parameters were measured with dedicated software (MM Oncology Lesion Scout, syngo.via Version 8.0; Siemens Healthineers). A whole-tumour volume of interest encompassing all PSMA-avid lesions was generated by semi-automatic segmentation using a 40% SUVmax threshold, excluding physiological uptake. Extracted metrics included RL-SUVmax, SUVmean, tPSMA-TV, PSMA-TVsal, PSMA-TV%, TLA, and hepatic SUVmax (3 cm spherical volume of interest in the right hepatic lobe) [23]. Quantitative extraction followed a predefined analysis workflow. Lesion count and volumetric/uptake measurements were recorded separately from the initial binary eligibility classification and were not disclosed as tabulated values during that classification, although overall lesion burden was inherently visible on the images.

### 2.3. [^177^Lu]Lu-PSMA Eligibility Criteria

Eligibility for [^177^Lu]Lu-PSMA RLT was determined retrospectively from PSMA PET/CT by two experienced nuclear medicine physicians (M.C. and G.P.), blinded to clinical and pathological data. The endpoint was imaging-defined eligibility according to VISION-like criteria, not complete clinical candidacy for therapy. Readers independently assessed all scans for uptake greater than hepatic background in measurable lesions and actively reviewed the CT component for measurable lesions with absent or insufficient PSMA. FDG PET was not performed [13]. Before consensus, the two readers agreed on 68 of the 76 scans (observed agreement: 89.5%; Cohen’s κ = 0.79, 95% CI: 0.65–0.93). The 8 discordant cases were adjudicated by a third physician (C.M.I.) in accordance with EANM/SNMMI guidance [13,17]. This PET-based classification served as the reference-standard outcome for all analyses.

### 2.4. Statistical Analysis

Right-skewed variables (PSA, tPSMA-TV, TLA, RL-SUVmax, and PSMA-TVsal) were log(1 + x)-transformed; other continuous predictors were analysed on their original scale, and categorical predictors were encoded with clinically meaningful reference categories. Three multivariable logistic regression models were developed—clinical, PSMA PET, and combined—using bidirectional Akaike information criterion-based stepwise selection, preceded by iterative variance inflation factor (VIF) pruning (threshold 5) to limit multicollinearity. Discrimination was assessed by receiver operating characteristic (ROC) and precision–recall area under the curve (AUC) with DeLong’s test, overall accuracy determined by Brier score and log-loss, and calibration by calibration-in-the-large and calibration slope. Internal validation used 500 bootstrap replicates, repeating the entire modelling strategy—including VIF pruning and stepwise selection—within each resample, with optimism-corrected estimates and patient-level out-of-bag (OOB) predictions. Nomograms were derived from the final models fitted on the full dataset. Analyses were performed in R 4.5.0 (R Foundation for Statistical Computing). Bidirectional AIC-based stepwise selection was used as the primary specification because the objective of this analysis was to characterise associations between candidate variables and PSMA PET-defined eligibility. Firth penalisation has been used to address the small-sample bias of maximum likelihood estimation, and was therefore applied for sensitivity analysis (Supplementary Materials). Because stepwise selection is unstable at this sample size, the entire procedure, including variance inflation pruning, was repeated within every bootstrap resample, so that the internally validated estimates were not inflated by selection, and the resulting selection frequencies are reported.

Inter-reader agreement for the binary eligibility classification before consensus was quantified using Cohen’s kappa with its 95% confidence interval.

During the preparation of this manuscript, the authors did not use any generative artificial intelligence tools.

## 3. Results

### 3.1. Study Population and Baseline Characteristics

**Figure 1:**
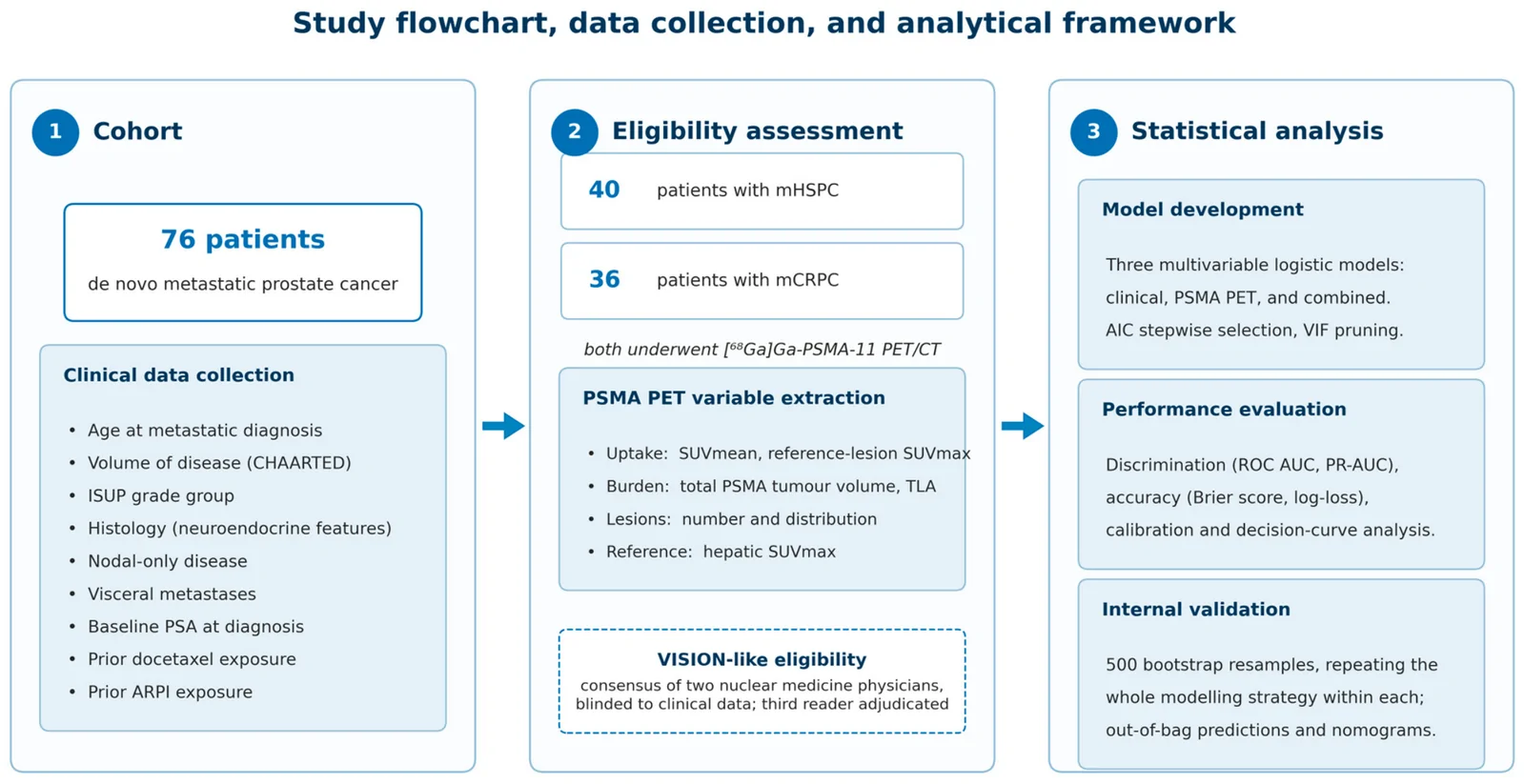
Study flowchart, data collection, and analytical framework.

The cohort comprised 76 men with de novo metastatic prostate cancer who underwent PSMA PET/CT in the mHSPC (*n* = 40) or mCRPC (*n* = 36) setting; the study flow and analytical framework are summarised in Figure 1. Forty-three patients (56.6%) were judged eligible for [^177^Lu]Lu-PSMA RLT; the remaining 33 (43.4%) were ineligible because of insufficient uptake, heterogeneous disease, or PSMA-negative measurable lesions. These categories describe imaging eligibility only and not final multidisciplinary treatment candidacy.

Age and ISUP grade did not differ significantly between groups (Table 1, Figure S1). Eligible patients had a substantially higher disease burden both at initial metastatic presentation and at PSMA PET/CT: 86.0% versus 42.4% had high-volume disease at conventional staging (*p* < 0.001), and 83.7% versus 27.2% had more than 10 PSMA-positive lesions. Eligible patients also showed higher SUVmean (15.7 versus 10.3, *p* < 0.001), RL-SUVmax (*p* = 0.02), and TLA (*p* = 0.03); more extensive nodal involvement (*p* = 0.02); and near-universal bone metastases (97.7% versus 78.8%, *p* = 0.02) (Table 1).

### 3.2. Univariable Predictors of [^177^Lu]Lu-PSMA Eligibility

**Figure 2:**
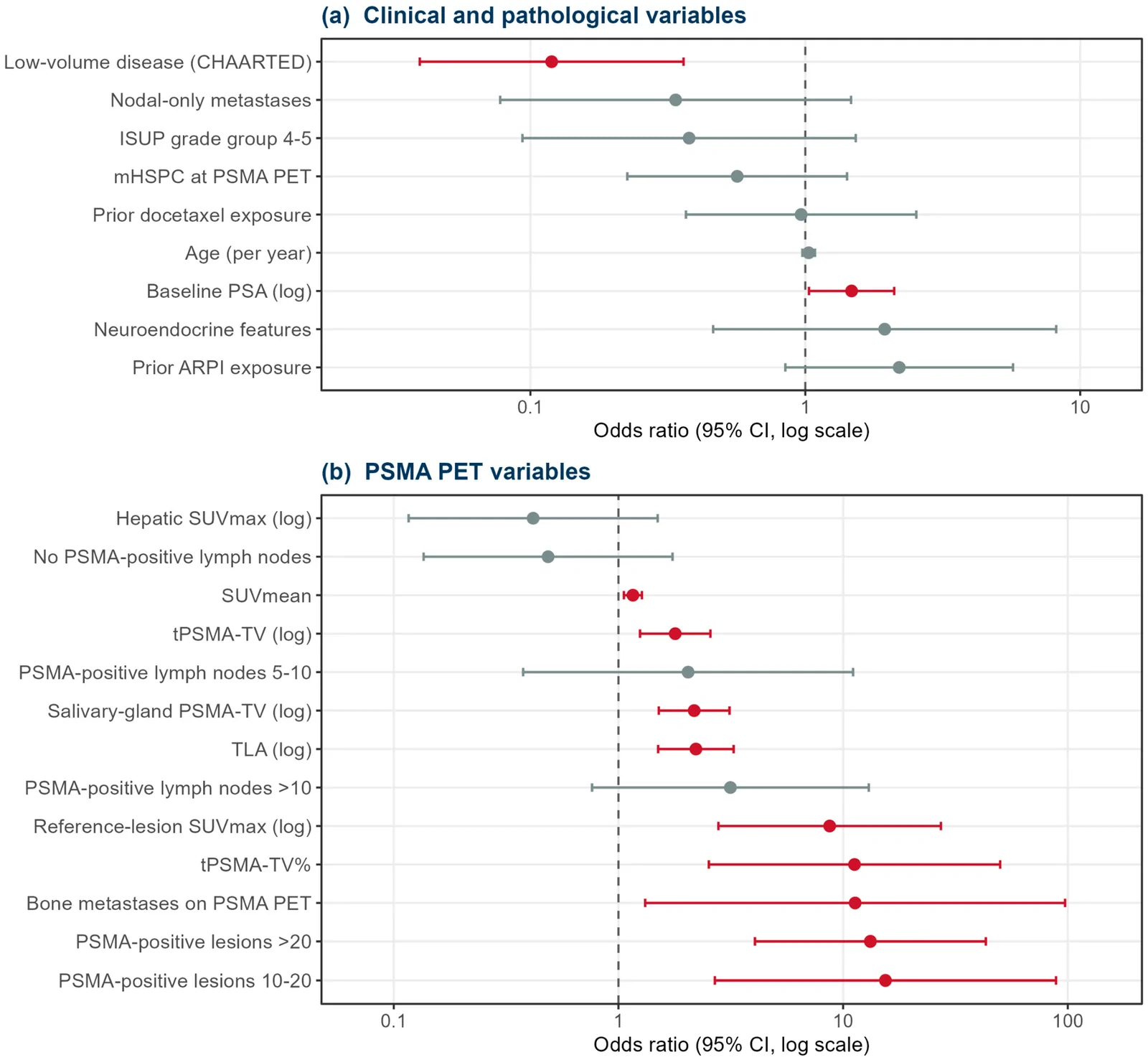
Forest plot of odds ratios for (**a**) clinical and (**b**) PSMA PET predictors of [^177^Lu]Lu-PSMA eligibility (definitions of the variables are shown in Table 2). Red markers indicate associations with 95% CI excluding 1 (nominally significant); grey markers indicate non-significant associations.

On univariable analysis, PSMA PET tumour burden metrics were the strongest predictors of eligibility (Figure 2, Table 2). More than 20 PSMA-positive lesions markedly increased the odds of eligibility versus fewer than 10 (odds ratio [OR]: 13.2; 95% CI: 4.29–46.6; *p* < 0.001), as did higher RL-SUVmax (OR per log-unit: 8.71; 95% CI: 3.10–31.1; *p* < 0.001). Volumetric parameters showed consistent positive associations (TLA OR: 2.21, 95% CI: 1.55–3.40; PSMA-TVsal OR: 2.17, 95% CI: 1.56–3.24; both *p* < 0.001). Among clinical variables, low-volume disease was associated with reduced eligibility (OR: 0.12; 95% CI: 0.04–0.34; *p* < 0.001), whereas bone metastases (OR: 11.3; 95% CI: 1.86–218; *p* = 0.03) and higher baseline PSA (OR per log-unit: 1.47; 95% CI: 1.06–2.18; *p* = 0.03) increased it. The inter-correlations used to inform variable selection are shown in Figure S2.

### 3.3. Multivariable Logistic Regression Models for Predicting [^177^Lu]Lu-PSMA Treatment Eligibility

**Figure 3:**
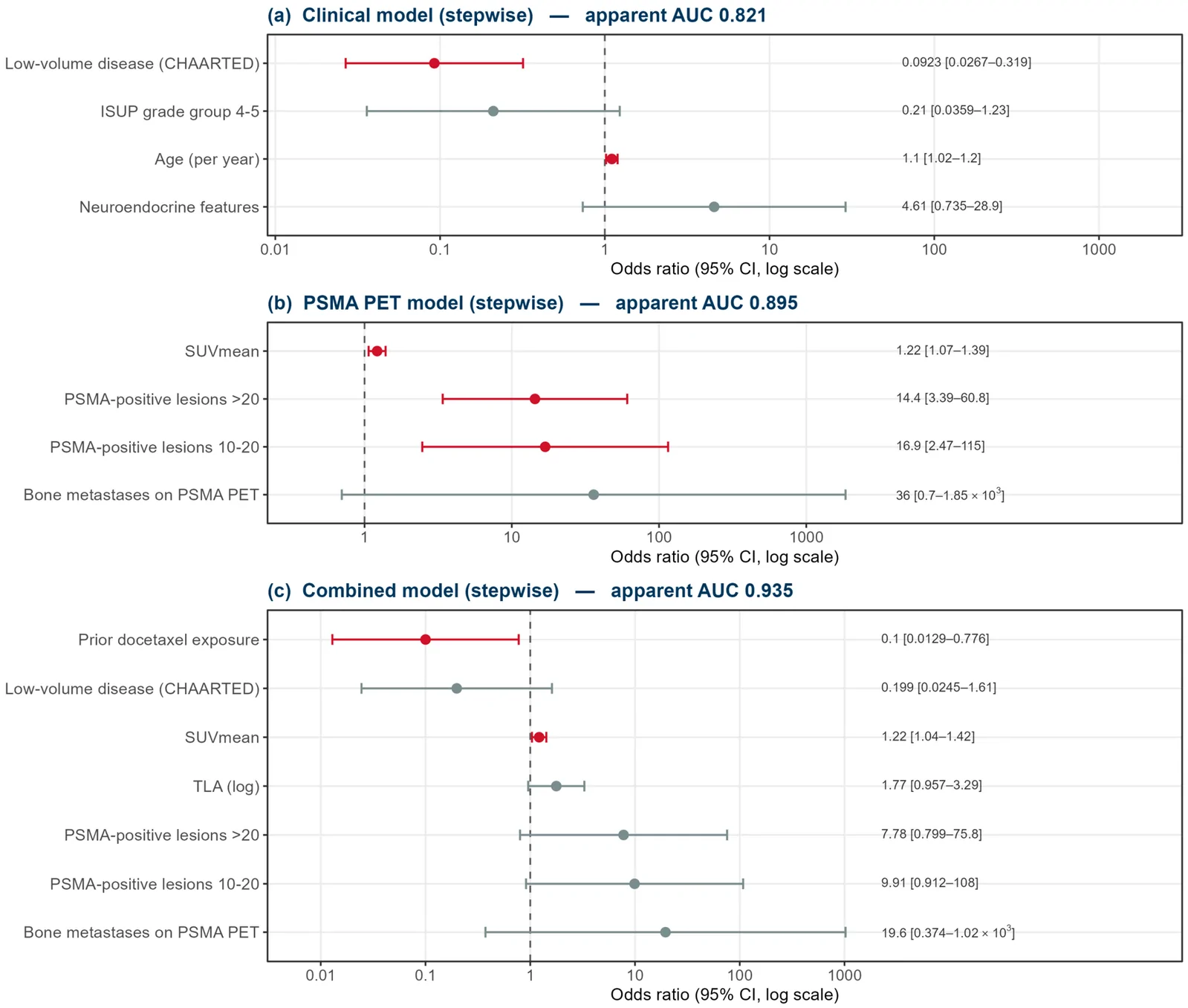
Forest plots of adjusted odds ratios from multivariable logistic regression models predicting [177Lu]Lu-PSMA treatment eligibility: (**a**) clinical model; (**b**) PSMA PET model; (**c**) combined clinical and PSMA PET model. Red markers indicate associations with 95% CIs excluding 1 (nominally significant), whereas grey markers indicate non-significant associations.

Stepwise selection produced three parsimonious models (Supplementary Result S1, Figure 3, Table 3). The clinical model retained age, volume of disease, ISUP grade, and adenocarcinoma histology (Figure 3a; apparent, i.e., optimistic, AUC of 0.821; internally validated out-of-bag AUC of 0.731), with age and disease volume as independent predictors (*p* < 0.02). The PET model retained PSMA-positive lesion count, SUVmean, and bone metastases (Figure 3b; apparent AUC of 0.895; internally validated out-of-bag AUC of 0.817), which were all independent except bone metastases (*p* < 0.004). The combined model retained volume of disease, prior docetaxel exposure, SUVmean, lesion count, bone metastases, and TLA (Figure 3c; apparent AUC of 0.935; internally validated out-of-bag AUC of 0.829, the largest apparent-to-validated reduction in the three models (0.106)), with prior docetaxel exposure and SUVmean as the only independent predictors (*p* < 0.03). Because the combined model retains several overlapping measures of PSMA-avid disease burden, its coefficients should be read collectively rather than as biologically independent effects; variance inflation pruning reduces but does not remove residual collinearity.

### 3.4. Model Validation

**Figure 4:**
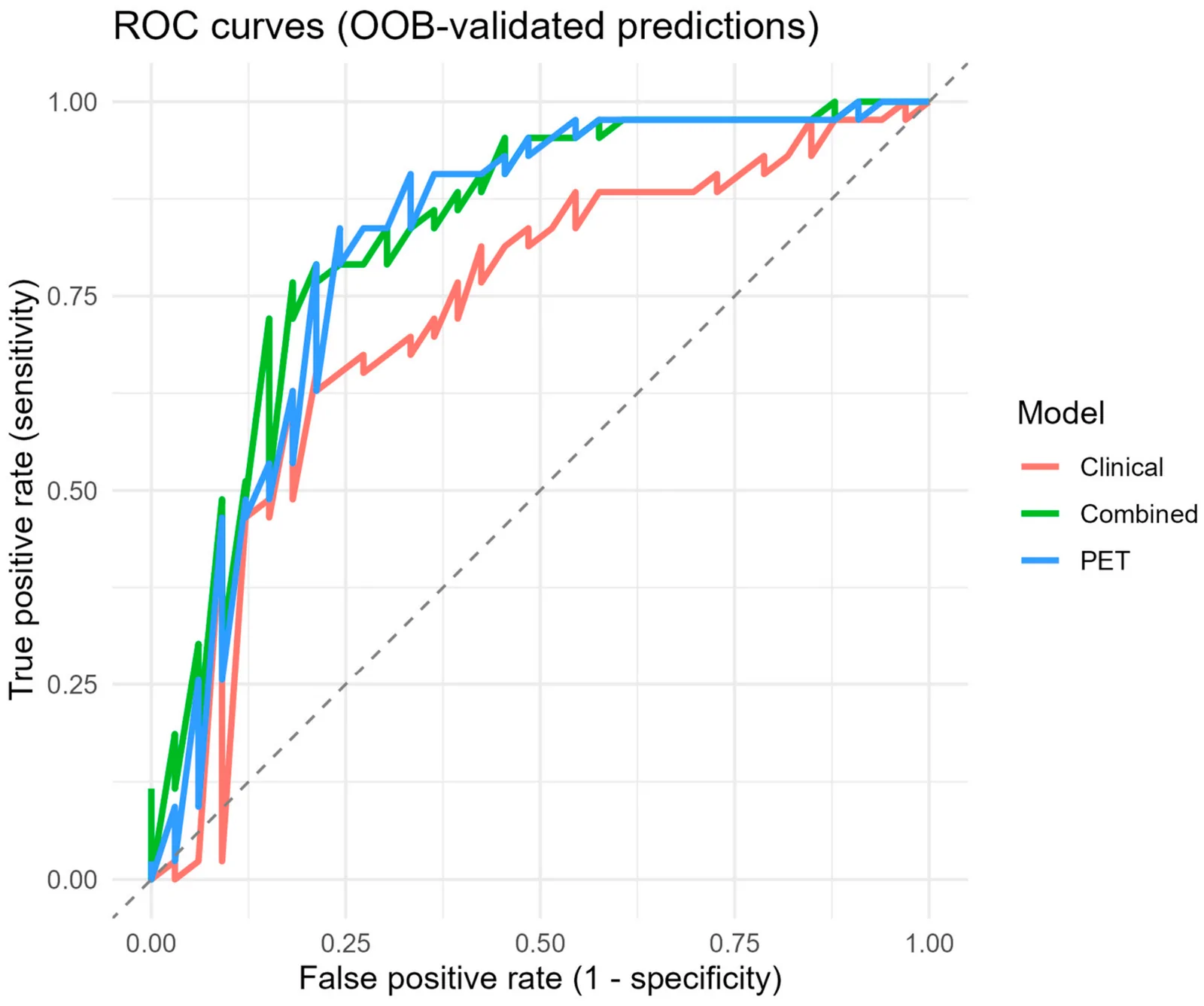
Out-of-bag ROC curves for the stepwise models predicting [^177^Lu]Lu-PSMA treatment eligibility from 500 bootstrap resamples (OOB AUCs are 0.731 for clinical model, 0.817 for PET model, and 0.829 for combined model). The dashed diagonal line represents the reference line for a non-discriminating classifier (AUC = 0.5).

**Figure 5:**
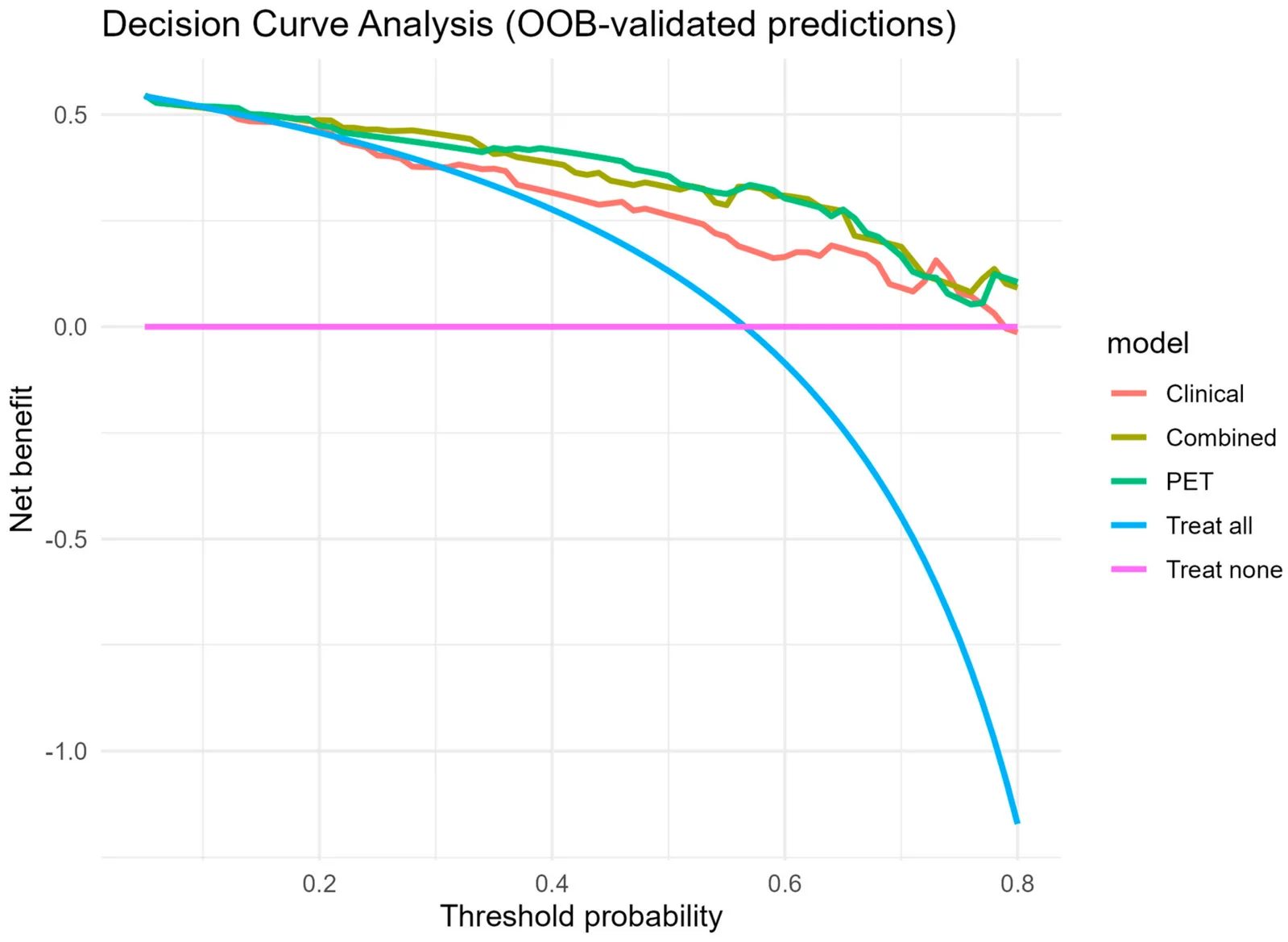
Decision curve analysis using internally validated out-of-bag predictions of the stepwise models for predicting [^177^Lu]Lu-PSMA treatment eligibility.

On internal validation (500 OOB bootstrap resamples), discrimination increased from the clinical model (OOB AUC of 0.731) to the PET model (0.817) and combined model (0.829) (Figure 4, Table S1). Pairwise differences were not significant between the combined and PET models (ΔAUC of 0.012, *p* = 0.62) or between the PET and clinical models (ΔAUC of 0.087, *p* = 0.20); the combined model showed borderline improvement over the clinical model (ΔAUC of 0.099, *p* = 0.08). PET-based models had lower probabilistic error than the clinical model (Brier: 0.16 versus 0.21), with the combined model showing the most favourable calibration (Figure S3). Decision curve analysis confirmed positive net benefit for all three models across a broad range of thresholds, with PET-based models providing the strongest net benefit and the combined model adding only a small, threshold-dependent advantage over the PET model (Figure 5, Table S2). Apparent and internally validated performance of the three stepwise models is presented in Table 4. Table 5 shows variable-selection stability across bootstrap resampling.

Taken together, PET-derived predictors carried most of the reproducible signal, with the combined model adding only a small gain in calibration rather than discrimination. The PET model therefore offered the best balance of validated performance, clinical utility, and parsimony, and was selected as the preferred specification; the clinical model, although superior to default strategies, was insufficient as a stand-alone tool when PET data were available. Variable-selection stability across resamples is summarised in Table S3. To support exploratory, prototype-level use, we derived nomograms for all three models; all three nomograms are provided in the Supplementary Materials (Figures S4–S8).

## 4. Discussion

In this exploratory study of 76 men with de novo metastatic prostate cancer, 56.6% met VISION-like PSMA PET criteria for [^177^Lu]Lu-PSMA. Measures of uptake and lesion burden were the most consistent correlates of the imaging-defined eligibility endpoint (Table S4). OOB discrimination was numerically higher for PET-based than for clinical-only modelling, but pairwise differences were not statistically significant, and the larger apparent-to-OOB reduction in the combined model indicated substantial optimism (Tables S5–S7). The results therefore support quantitative formalisation of expert PET interpretation rather than a validated prediction tool.

Nevertheless, the combined model provided clinically relevant insight: after accounting for both imaging and clinical factors, SUVmean remained independently associated with a higher probability of candidacy, whereas prior docetaxel exposure showed the opposite association (Table S8). These findings confirm that VISION-like eligibility is driven primarily by the extent and intensity of PSMA-avid disease. At the same time, the independent association between prior docetaxel exposure and lower candidacy is hypothesis-generating and raises a clinically relevant question: whether exposure to specific systemic treatments may modify the probability of subsequent [^177^Lu]Lu-PSMA eligibility, with potential implications for treatment sequencing, although this association is currently still defined as exploratory and statistically fragile.

Among PSMA PET predictors, tumour burden and tracer avidity were the strongest determinants of eligibility. Higher SUVmean was a key independent predictor, consistent with the need for sufficient PSMA expression for effective targeting [13,18,23], while lesion count and TLA captured complementary information on disease extent. These findings reinforce the biological premise of trial-based selection, in which adequate target expression across measurable disease underpins the response [6,28]. Higher hepatic uptake tended to reduce eligibility, likely by lowering lesion-to-liver contrast, in line with evidence that tracers with predominant hepatic excretion may reduce the eligible proportion [29].

Clinical variables retained in exploratory models included CHAARTED disease volume, presence of neuroendocrine differentiation at histology, and ISUP grade [30,31,32,33,34]. CHAARTED volume was assigned from conventional imaging at initial metastatic presentation, whereas PSMA lesion count was obtained from PET, sometimes later in the disease course. These constructs overlap but are not identical. Although higher tumour grade is generally associated with lower PSMA expression [19], the higher eligibility of patients with pure adenocarcinoma underscores the importance of preserved differentiation, since neuroendocrine or other dedifferentiated and non-adenocarcinoma variants typically show reduced PSMA expression [35]. Low-volume disease at mHSPC diagnosis was associated with markedly lower eligibility (OR 0.09), likely reflecting lower PSMA-avid tumour burden and fewer lesions fulfilling the diffuse, high-burden, PSMA-positive phenotype selected by VISION-like criteria. This distinction is important because these criteria capture sufficiently intense and widespread PSMA expression across measurable disease, rather than tumour aggressiveness alone.

The low-volume association should not be interpreted simply as a biological effect or as a restatement that fewer lesions are present. Interval treatment and disease evolution may also weaken the correspondence between baseline CHAARTED volume and later PSMA PET findings. In a focused analysis, this association was substantially attenuated after adjustment for the number of PSMA-positive lesions (adjusted odds ratio: 0.45, 95% CI: 0.11–1.91, *p* = 0.28; unadjusted odds ratio: 0.12, *p* < 0.001), indicating that the apparent effect of conventional-imaging disease volume is largely explained by PSMA-positive lesion burden rather than acting independently of it.

Other clinical associations were less robust and should be considered exploratory. Older age was modestly associated with candidacy in clinical modelling (OR 1.10), but this may reflect residual confounding by disease burden or treatment selection rather than a direct biological effect, although age-related differences in tumour biology cannot be excluded. Disease state at imaging (mHSPC vs. mCRPC) was not associated with candidacy, suggesting no clear setting-dependent loss of VISION-like eligibility in this cohort. This finding is clinically relevant because a higher eligibility rate in mHSPC would raise concern that delaying [^177^Lu]Lu-PSMA until mCRPC could exclude some patients from later treatment; prospective longitudinal studies are needed to test this hypothesis. Visceral disease showed discordant signals: visceral metastases at initial presentation were associated with a higher probability of candidacy on univariable analysis, whereas visceral involvement on PSMA PET was not. This likely reflects timing differences between baseline staging and functional reassessment, small numbers, and selection effects, rather than a true independent association between visceral spread and higher likelihood of meeting VISION-like criteria. Visceral metastases should not preclude [^177^Lu]Lu-PSMA consideration when uptake is sufficient; candidacy should remain based on demonstrated PSMA expression and the absence of discordant PSMA-negative disease [36,37,38,39]. In addition, prior docetaxel exposure was nearly equally distributed between eligible and ineligible groups and should be regarded as hypothesis-generating. Prior docetaxel may mark treatment sequencing, disease duration, prior ARPI exposure, tumour biology, or clinical selection rather than a direct effect on PSMA expression. Because paired pre- and post-docetaxel imaging was unavailable, the present cross-sectional analysis cannot determine whether docetaxel changes PSMA expression or later treatment access. Beyond the effect of individual clinical variables, the performance of the clinical model is also informative. Despite excluding all PSMA PET/CT metrics, it retained internally validated discrimination with an out-of-bag AUC of 0.731, suggesting that combined clinical features capture profiles associated with different probabilities of PET-defined candidacy. This may reflect underlying differences in tumour burden, biology, prior treatment exposure, and PSMA expression or distribution. If externally validated, such a model could support pre-imaging prioritisation and help guide when repeat PSMA PET/CT assessment is most informative, particularly in settings where molecular imaging resources are limited, consistent with EANM/SNMMI guidance on rational allocation of molecular imaging [13].

Few studies have directly modelled [^177^Lu]Lu-PSMA eligibility using both clinical and PSMA PET parameters. Unlike nomograms developed to predict outcomes after treatment [40], our models address eligibility itself: an operational, context-dependent endpoint shaped by trial criteria, imaging interpretation, and local practice. The PET model partly reproduces features used by readers when assigning eligibility. Its role is therefore criterion-related formalisation of expert imaging assessment, not independent prediction of treatment benefit or complete clinical candidacy. Actual treatment candidacy also depends on performance status, marrow and organ function, comorbidities, previous therapies, expected benefit, multidisciplinary review, and patient preference. This is clinically relevant because candidacy assessment determines access to treatment and remains vulnerable to operator- and centre-dependent variability. We were able to quantify this directly: before consensus, two experienced readers applying identical VISION-like criteria to the same images disagreed on 8 of 76 scans (observed agreement: 89.5%; κ = 0.79), so approximately one case in ten required adjudication even within a single centre. This supports the premise that eligibility assessment, although performed by experts, benefits from explicit and quantifiable imaging determinants. Accordingly, these models should be viewed as decision-support tools, not prognostic or treatment-response models. Their value lies in making expert assessment more explicit, reproducible, and auditable, supporting standardised VISION-like candidacy and more comparable patient selection across centres. Before clinical implementation; however, they require external multicentre validation, including comparisons with eligibility assessments by independent experts from different institutions. Future work should integrate PSMA PET phenotypes with molecular and multi-omic markers. Driver alterations, genomic instability, tumour-microenvironment signatures, and multimodal diagnostic approaches may explain biological heterogeneity not captured by PSMA uptake alone and may permit more precise, disease-state-specific stratification [24,25,26].

By including PSMA PET/CT performed in both mHSPC and mCRPC, our study addresses an increasingly relevant context, as PSMA-targeted RLT is being evaluated earlier in the prostate cancer disease course, including in mHSPC and earlier-line mCRPC settings [9,10]. In our cohort, disease state at imaging did not substantially influence VISION-like candidacy, suggesting neither a clear setting-dependent loss of eligibility nor major differences in the PET-derived determinants underlying eligibility across these settings. Subgroup analyses are descriptive because the strata are small and cannot validate separate models (Tables S9 and S10). In addition, all participants had de novo metastatic disease; the findings may not apply to patients with metachronous metastases and different treatment histories. However, because trial-specific selection criteria vary and our analysis was not longitudinal within the same patients, these findings should not be considered interchangeable with any single trial framework.

This study has several limitations. It was retrospective, single-centre, and limited in sample size, resulting in a low number of events per variable (33 events for six predictors in the combined model). The reduction from apparent to OOB performance indicates optimism, particularly for the combined model. Repeating the complete modelling procedure within bootstrap resamples addresses some selection-induced optimism but external validation in a larger, independent cohort or a clinical-trial dataset is essential before any clinical application, and we regard this as the principal limitation. The endpoint was expert consensus eligibility according to VISION-like PSMA PET criteria, not actual multidisciplinary candidacy for radioligand therapy or treatment benefit. Because PET-derived uptake and burden measures were modelled against a PET-defined endpoint, incorporation bias was unavoidable; the PET model should be regarded as a quantitative formalisation of expert imaging assessment. Although imaging was standardised, differences in scanners, reconstruction, and segmentation thresholds may affect the reproducibility of PSMA PET metrics [34].

The cohort combined mHSPC and mCRPC and included only de novo metastatic disease, limiting generalisability. Treatment-history coefficients, particularly prior docetaxel exposure, may reflect sparse data, confounding by indication, treatment sequencing, and unmeasured biology. Finally, prototype nomograms require external validation and recalibration and should not be used for clinical decisions.

## 5. Conclusions

In this exploratory cohort of men with de novo metastatic prostate cancer, VISION-like PSMA PET-defined eligibility was principally associated with imaging measures of disease extent and target expression, particularly PSMA-positive lesion count and SUVmean. PET-based modelling captured most of the reproducible eligibility signal, whereas clinical variables added only limited value.

Two findings merit further study. The clinical-only model could, if externally validated, help prioritise access to PSMA PET/CT where imaging capacity is limited, without replacing imaging-based assessment. Men previously treated with docetaxel appeared less likely to meet eligibility criteria, but this signal was inconsistent and should be read as a question to be tested rather than an established effect; it nonetheless raises the clinically relevant possibility that the order in which systemic treatments are given may influence later access to [^177^Lu]Lu-PSMA RLT. Answering that question will require imaging obtained before and after chemotherapy, and the present data should not be read as evidence that chemotherapy reduces PSMA expression.

External multicentre validation, including comparison with eligibility assessments by independent experts across institutions, is required before these models or the accompanying nomograms can be considered for clinical use.

## Figures and Tables

**Table 1:** Clinical and demographic characteristics of included patients and PSMA-PET parameters stratified by [^177^Lu]Lu-PSMA candidacy.

Variable	Total (*n* = 76)	Not Candidate (*n* = 33)	Candidate (*n* = 43)	*p*-Value *
Age at mPC diagnosis (years, mean ± SD)	72.54 ± 8.85	71.31 ± 6.49	73.48 ± 10.28	0.293
Baseline PSA at mPC diagnosis (ng/mL, mean ± SD)	206.29 ± 668.03	73.95 ± 141.75	307.85 ± 870.21	0.131
High-volume disease (CHAARTED criteria), *n* (%)	51 (67.1)	14 (42.4)	37 (86.0)	<0.001
Nodal-only disease, *n* (%)	9 (11.8)	6 (18.2)	3 (7.0)	0.254
Visceral metastases present, *n* (%)	18 (23.7)	4 (12.1)	14 (32.6)	0.071
ISUP grade groups 4–5, *n* (%)	64 (84.2)	30 (90.9)	34 (79.1)	0.278
Non-neuroendocrine adenocarcinoma, *n* (%)	66 (86.8)	30 (90.9)	36 (83.7)	0.564
mHSPC patients at time of PET, *n* (%)	40 (52.6)	20 (60.6)	20 (46.5)	0.323
Prior ARPI exposure, *n* (%)	31 (40.8)	10 (30.3)	21 (48.8)	0.163
Prior docetaxel exposure, *n* (%)	25 (32.9)	11 (33.3)	14 (32.6)	1.00
SUVmax of RL (mean ± SD)	39.56 ± 49.73	24.12 ± 13.47	51.41 ± 62.82	0.017
Total PSMA tumour volume (mL, mean ± SD)	92.53 ± 138.01	65.12 ± 134.38	113.57 ± 138.59	0.130
TLA (mean ± SD)	1211.02 ± 1991.49	661.90 ± 1973.03	1632.44 ± 1922.88	0.034
SUVmean (mean ± SD)	13.32 ± 6.89	10.26 ± 5.98	15.66 ± 6.68	<0.001
Liver SUVmax (mean ± SD)	14.55 ± 5.73	15.86 ± 7.15	13.55 ± 4.16	0.081
Baseline PSA, mean of log ± SD	3.77 ± 1.51	3.34 ± 1.29	4.11 ± 1.60	0.026
SUVmax of RL, mean of log ± SD	3.44 ± 0.67	3.08 ± 0.57	3.71 ± 0.60	<0.001
tPSMA-TV, mean of log ± SD	3.52 ± 1.53	2.85 ± 1.61	4.04 ± 1.26	0.001
TLA, mean of log ± SD	5.88 ± 1.73	4.85 ± 1.68	6.67 ± 1.31	<0.001
Hepatic SUVmax, mean of log ± SD	2.68 ± 0.38	2.74 ± 0.41	2.63 ± 0.35	0.177
Number of PSMA-positive lesions, *n* (%)				<0.001
<10	31 (40.8%)	24 (72.7%)	7 (16.3%)	
10–20	11 (14.5)	2 (6.1%)	9 (20.9%)	
>20	34 (44.7)	7 (21.2%)	27 (62.8%)	
Number of PSMA-positive lymph nodes, *n* (%)				0.017
0	28 (36.8%)	18 (54.5%)	10 (23.3%)	
<5	15 (19.7%)	7 (21.2%)	8 (18.6%)	
5–10	10 (13.2%)	3 (9.1%)	7 (16.3%)	
>10	23 (30.3%)	5 (15.2%)	18 (41.9%)	
Bone metastases on PSMA-PET, *n* (%)	68 (89.5%)	26 (78.8%)	42 (97.7%)	0.022
Visceral metastases on PSMA-PET, *n* (%)	21 (27.6%)	8 (24.2%)	13 (30.2%)	0.749

ARPI, androgen receptor pathway inhibitor; ISUP, International Society of Urological Pathology; LV, low-volume; mHSPC, metastatic hormone-sensitive prostate cancer; mPC, metastatic prostate cancer; tPSMA-TV, total PSMA tumour volume; RL, reference lesion; SD, standard deviation; SUVmax, standardised uptake value maximum; SUV mean, standardised uptake value mean; TLA, total lesion activity; * comparing “not candidate” versus “candidate”.

**Table 2:** Univariable logistic regression analyses for predicting [^177^Lu]Lu-PSMA eligibility.

Predictor	Unit or Levels	Reference Level ^1^	OR	95% CI	*p*-Value
Age at mPC diagnosis	Year	unit	1.03	[0.977–1.09]	0.29
Volume of disease according to CHAARTED criteria	High, low	High	0.12	[0.0368–0.344]	<0.001
Nodal-only metastatic disease	No, yes	No	0.34	[0.0666–1.39]	0.15
Visceral metastases present	No, yes	No	3.50	[1.10–13.5]	0.045
ISUP grade groups 4–5	<4, ≥5	<4	0.38	[0.0784–1.40]	0.17
Presence of neuroendocrine features at histology	Pure Adenocarcinoma	Presence of neuroendocrine features	1.94	[0.494–9.63]	0.36
mHSPC or mCRPC at time of PET	mCRPC, mHSPC	mCRPC	0.57	[0.222–1.41]	0.22
Prior ARPI exposure	No, yes	No	2.20	[0.86–5.86]	0.11
Prior docetaxel exposure	No, yes	No	0.97	[0.368–2.57]	0.94
Baseline PSA	log ng/mL	log unit	1.47	[1.06–2.18]	0.033
SUV mean	unit	unit	1.16	[1.07–1.28]	0.002
SUVmax of RL	log unit	log unit	8.71	[3.10–31.1]	<0.001
Hepatic SUVmax	log unit	log unit	0.42	[0.107–1.43]	0.18
Salivary gland PSMA-TV	log unit	log unit	2.17	[1.56–3.24]	<0.001
TLA	log unit	unit	2.21	[1.55–3.40]	<0.001
PSMA-TV percentage	unit	unit	11.2	[2.77–56.8]	0.002
tPSMA-TV	log unit	log unit	1.79	[1.27–2.64]	0.002
Number of PSMA-positive lesions	<10, >20	<10	13.2	[4.29–46.6]	<0.001
Number of PSMA-positive lesions	<10, 10–20	<10	15.4	[3.13–119]	0.002
Bone metastases at PSMA-PET	No, yes	No	11.3	[1.86–218.0]	0.027
Number of PSMA-positive lymph nodes	<5, >10	<5	3.15	[0.78–13.8]	0.11
Number of PSMA-positive lymph nodes	<5, 5–10	<5	2.04	[0.392–12.5]	0.41
Presence of PSMA-positive lymph nodes	Present, absent	Present	0.49	[0.132–1.73]	0.27
Visceral metastases at PSMA-PET	No, yes	No	1.35	[0.49–3.91]	0.56

^1^ unit, analysed as a continuous variable. The OR refers to the increase in one unit. ARPI, androgen receptor pathway inhibitor; ISUP, International Society of Urological Pathology; mHSPC, metastatic hormone-sensitive prostate cancer; mPC, metastatic prostate cancer; tPSMA-TV, total PSMA tumour volume; RL, reference lesion; SUVmax, standardised uptake value maximum; SUVmean, standardised uptake value mean; TLA, total lesion activity.

**Table 3:** Exact odds ratios and 95% confidence intervals for the combined stepwise model shown in Figure 3c.

Predictor	Odds Ratio	95% CI	*p*-Value
Low-volume disease (CHAARTED)	0.199	0.02449 to 1.612	0.130
Prior docetaxel exposure	0.100	0.01291 to 0.7762	0.028
SUVmean	1.216	1.039 to 1.424	0.015
PSMA-positive lesions > 20	7.780	0.799 to 75.75	0.077
PSMA-positive lesions 10–20	9.911	0.9117 to 107.7	0.060
Bone metastases on PSMA PET	19.555	0.3736 to 1023	0.141
TLA (log)	1.773	0.9566 to 3.286	0.069

**Table 4:** Apparent and internally validated performance of the three stepwise models.

Model	AUC Apparent	AUC Validated	Optimism	PR-AUC Apparent	PR-AUC Validated	Brier Validated	Log-Loss Validated	Calibration Slope
Clinical	0.821	0.731	0.090	0.854	0.743	0.209	0.616	0.704
PSMA PET	0.895	0.817	0.078	0.902	0.796	0.165	0.528	0.737
Combined	0.935	0.829	0.106	0.945	0.839	0.157	0.492	0.915

Apparent values are in-sample and therefore optimistic; validated values are out-of-bag estimates obtained from 500 bootstrap resamples in which the entire modelling strategy, including variance inflation pruning and stepwise selection, was repeated within each resample. Optimism is calculated as the apparent minus the validated AUC. A calibration slope below 1 indicates overfitting, with 1 denoting perfect calibration. Abbreviations: AUC, area under the receiver operating characteristic curve; OOB, out-of-bag; PET, positron emission tomography; PR-AUC, area under the precision–recall curve; PSMA, prostate-specific membrane antigen.

**Table 5:** Variable-selection stability across bootstrap resampling.

Predictor	Clinical	PSMA PET	Combined
Age at metastatic diagnosis	89%	-	52%
Bone metastases on PSMA PET	-	74%	52%
Disease state at PSMA PET	25%	-	27%
Hepatic SUVmax (log)	-	12%	52%
Histology (neuroendocrine features)	71%	-	65%
ISUP grade group	67%	-	66%
Nodal-only disease	43%	-	36%
Number of PSMA-positive lesions	-	80%	59%
Number of PSMA-positive lymph nodes	-	24%	39%
Prior ARPI exposure	-	-	26%
Prior docetaxel exposure	-	-	28%
Reference-lesion SUVmax (log)	-	22%	50%
SUVmean	-	74%	39%
Salivary gland PSMA-TV (log)	-	40%	43%
Total lesion activity (log)	-	36%	44%
Volume of disease (CHAARTED)	94%	-	70%
tPSMA-TV%	-	22%	45%

Values are the percentages of the 500 bootstrap resamples in which each candidate predictor was retained by the stepwise selection procedure. A dash indicates that the variable was not a candidate for that model. Variables marked (log) were analysed after log(1 + x) transformation. Predictors retained in only a minority of resamples should be regarded as unstable, and their individual coefficients interpreted with caution. Abbreviations: ARPI, androgen receptor pathway inhibitor; CHAARTED, Chemohormonal Therapy versus Androgen Ablation Randomized Trial for Extensive Disease in Prostate Cancer; ISUP, International Society of Urological Pathology; PET, positron emission tomography; PSMA, prostate-specific membrane antigen; PSMA-TV, PSMA tumour volume; SUVmax, maximum standardised uptake value; SUVmean, mean standardised uptake value; tPSMA-TV, total PSMA tumour volume.

## Data Availability

The data supporting the findings of this study are available from the corresponding author upon reasonable request.

## Supplementary Materials

The following supporting information can be downloaded at https://www.mdpi.com/article/10.3390/biomedicines14081818/s1: Section S1: Statistical Analysis; Table S1: Internal validation, calibration, discrimination, and classification performance of stepwise clinical, PSMA PET, and combined prediction models; Table S2: Apples-to-apples OOB classification performance at fixed probability thresholds; Table S3: Variable-selection stability for the stepwise clinical, PSMA PET and combined prediction models across bootstrap resamples; Table S4: PSMA volumetric parameters relative to the salivary-gland uptake threshold, by [177Lu]Lu-PSMA eligibility; Table S5: Models based on clinical variables; Table S6: Models based on PSMA-PET parameters; Table S7: Models combining clinical variables and PSMA-PET parameters; Table S8: Firth penalised logistic regression of the combined stepwise model; Table S9: Descriptive eligibility rates and internally validated discrimination within the mHSPC and mCRPC subgroups; Table S10: Low-volume disease before and after adjustment for the number of PSMA-positive lesions; Result S1: Model development and performance; Result S2: Internal validation, classification profiles, and model comparison; Result S3: Sensitivity analyses for the prior-docetaxel coefficient; Result S4: Disease-state subgroups (mHSPC versus mCRPC); Result S5: CHAARTED volume adjusted for PSMA-positive lesion count; Figure S1: Distribution of key clinical, pathological, and PSMA PET parameters according to [^177^Lu]Lu-PSMA eligibility; Figure S2: Correlation matrix of clinical, pathological, and PSMA PET parameters; Figure S3: Calibration plots for the three stepwise multivariable models; Figure S4: Quantile–quantile plots of residuals; Figure S5: Nomogram of the clinical-only stepwise model; Figure S6: Nomogram of the combined stepwise model; Figure S7: Internally validated OOB performance summary across stepwise models. Figure S8: Nomogram of the PSMA PET stepwise model estimating the probability of eligibility for [177Lu]Lu-PSMA therapy in de novo metastatic prostate cancer.

## Abbreviations

The following abbreviations are used in this manuscript:

ARPI	Androgen receptor pathway inhibitor
AUC	Area under the curve
BIC	Bayesian information criterion
CT	Computed tomography
ISUP	International Society of Urological Pathology
mCRPC	Metastatic castration-resistant prostate cancer
mHSPC	Metastatic hormone-sensitive prostate cancer
tPSMA-TV	Total PSMA tumour volume
OOB	Out-of-bag
PET	Positron emission tomography
PSA	Prostate-specific antigen
PSMA	Prostate-specific membrane antigen
RL	Reference lesion
SUV	Standardised uptake value
TLA	Tumour lesion activity
