# Supplementary material for: Imaging and Clinical Correlates of [177Lu]Lu-PSMA PET-Defined Eligibility in De Novo Metastatic Prostate Cancer
Source: Biomedicines. 2026 Aug 13;14(8):1818. doi: 10.3390/biomedicines14081818 (PMC13509732; doi:10.3390/biomedicines14081818)
Supplement: Supplementary file 1 [file biomedicines-14-01818-s001.zip › biomedicines-4452203-supplementary.pdf]

International Journal of *Molecular Sciences*

Supplementary Materials

Imaging and Clinical Correlates of [^{177}Lu]Lu-PSMA PET-Defined Eligibility in De Novo Metastatic Prostate Cancer

Contents

Appendix S1. Statistical Analysis.

Table S1. Internal validation, calibration, discrimination, and classification performance of stepwise clinical, PSMA PET, and combined prediction models.

Table S2. Apples-to-apples OOB classification performance at fixed probability thresholds

Table S3. Variable selection stability for the stepwise Clinical, PSMA PET and combined prediction models across bootstrap resamples.

Table S4. PSMA volumetric parameters relative to the salivary-gland uptake threshold, by [^{177}Lu]Lu-PSMA eligibility.

Table S5. Models based on clinical variables.

Table S6. Models based on PSMA-PET parameters.

Table S7. Models combining clinical variables and PSMA-PET parameters.

Table S8. Firth penalised logistic regression of the combined stepwise model.

Table S9. Descriptive eligibility rates and internally validated discrimination within the mHSPC and mCRPC subgroups.

Table S10. Low-volume disease before and after adjustment for the number of PSMA-positive lesions.

Result S1 – Model Development and Performance

Result S2 - Internal Validation, Classification Profiles, and Model Comparison

Result S3 - Sensitivity analyses for the prior-docetaxel coefficient

Result S4 - Disease-state subgroups (mHSPC versus mCRPC)

Result S5 - CHAARTED volume adjusted for PSMA-positive lesion count

Figure S1. Distribution of key clinical, pathological, and PSMA PET parameters according to [^{177}Lu]Lu PSMA eligibility

Figure S2. Correlation matrix of clinical, pathological, and PSMA PET parameters

Figure S3. Calibration plots for the three stepwise multivariable models predicting [^{177}Lu]Lu-PSMA treatment eligibility using internally validated outofbag (OOB) predictions

Figure S4. Quantile–quantile plots of residuals for stepwise clinical, PSMA PET, and combined models

Figure S5. Nomogram of the clinical-only stepwise model estimating the probability of eligibility for [^{177}Lu]Lu PSMA therapy in de novo metastatic prostate cancer

Figure S6. Nomogram of the combined stepwise model estimating the probability of eligibility for [^{177}Lu]Lu-PSMA therapy in de novo metastatic prostate cancer.

Figure S7. Internally validated OOB performance summary across stepwise models

Figure S8. Nomogram of the PSMA PET stepwise model estimating the probability of eligibility for [^{177}Lu]Lu-PSMA therapy in de novo metastatic prostate cancer.

Section S1. Statistical Analysis

Data preprocessing

Continuous variables were inspected for distributional shape and potential leverage. Variables showing marked right-skewness were transformed using a log(1 + x) transformation to reduce the influence of extreme values and improve approximate linearity on the log-odds scale. Continuous variables without relevant skewness were analysed on their original scale. Categorical predictors were encoded as factors using clinically meaningful reference categories.

Model development

Three multivariable logistic regression models were developed with the binary outcome modelled using logistic regression (**glm**, binomial family). The clinical-only model included baseline clinical-pathological variables and treatment history to represent a pre-imaging triage scenario. The PET model included imaging-derived PSMA PET/TAC metrics only. The combined model included both clinical-pathological predictors and PET-derived variables.

To obtain parsimonious models, variable selection was performed using bidirectional stepwise selection based on Akaike's Information Criterion (AIC). To limit multicollinearity, variance inflation factors were assessed and variables with excessive collinearity were excluded via an iterative VIF-based pruning procedure prior to stepwise selection using a predefined VIF threshold of 5. Both the VIF-based pruning and the AIC stepwise selection were embedded within the internal validation resampling procedure, such that the entire modelling strategy was repeated from scratch within each resample to account for selection-induced optimism. As a measure of variable-selection stability, selection frequency across resamples was recorded for each candidate predictor.

Model performance metrics

Model performance was quantified in terms of discrimination, overall accuracy, and calibration. Discrimination was assessed using the area under the receiver operating characteristic curve (ROC AUC). Precision-recall performance was summarised using average precision (PR-AUC). Overall predictive accuracy was quantified using the Brier score and logarithmic loss (log-loss). Calibration was assessed using calibration-in-the-large (intercept) and calibration slope estimated by regressing the observed outcome on the model logit-transformed predicted probabilities, with a slope below 1 indicating potential overfitting.

Internal validation and uncertainty quantification

Internal validation was performed using bootstrap resampling. For each bootstrap replicate, the full modelling strategy was repeated including VIF-based collinearity pruning and AIC-based stepwise selection. Out-of-bag (OOB) predictions were generated for each individual from bootstrap replicates in which that individual was not included, and then aggregated across replicates to provide internally validated predicted probabilities for each patient. These aggregated OOB predictions were used to estimate internally validated OOB AUC, PR-AUC, Brier score, log-loss, and calibration measures.

Uncertainty around OOB performance metrics was quantified using nonparametric bootstrap confidence intervals based on 2000 patient-level resamples applied to the aggregated OOB predictions. No cross-validation procedures were used.

For each internally validated final stepwise model, a nomogram was subsequently derived to facilitate individual-level risk estimation and clinical interpretability.

Software and packages

All analyses were conducted in R (version 4.5.0). Data management used dplyr and tidyr. Logistic regression models were fitted using base R stats (glm, binomial family). Discrimination metrics were computed using pROC. Penalised regression sensitivity analyses, when used, relied on glmnet and Matrix. Decision curve analysis used rmda. Plots and figure assembly used ggplot2-ecosystem utilities including patchwork and gridExtra.

Table S1. Internal validation, calibration, discrimination, and classification performance of stepwise clinical, PSMA PET, and combined prediction models.

Metric	Clinical	PSMA PET	Combined
Apparent AUC	0.821	0.895	0.935
OOB AUC (internally validated)	0.731	0.817	0.829
AUC optimism (Apparent - OOB)	0.09	0.078	0.106
OOB Sensitivity (Youden threshold)	0.628	0.837	0.767
OOB Specificity (Youden threshold)	0.818	0.758	0.818
OOB Accuracy (Youden threshold)	0.711	0.803	0.789
OOB Brier score	0.208	0.164	0.164
OOB LogLoss	0.614	0.528	0.513
OOB Calibration intercept	0.09	0.08	0.1
OOB Calibration slope	0.775	0.723	0.815
OOB ECE (10 bins)	0.162	0.12	0.101
Delta AUC vs Clinical (OOB, DeLong)		0.087	0.099
p-value vs Clinical (OOB, DeLong)		0.203	0.081
Delta AUC vs PSMA PET (OOB, DeLong)	-0.087		0.012
p-value vs PSMA PET (OOB, DeLong)	0.203		0.624

Abbreviations. AUC area under the receiver-operating characteristic curve; ROC receiver-operating characteristic; OOB out-of-bag; PET positron emission tomography; PSMA prostate-specific membrane antigen; [^{177}Lu]Lu-PSMA lutetium177–labelled PSMA-targeted radioligand; ECE expected calibration error; ΔAUC difference in AUC; Se sensitivity; Sp specificity; Acc accuracy; PPV positive predictive value; NPV negative predictive value.

Apparent AUCs represent in-sample discrimination, whereas OOB AUCs reflect discrimination after internal validation based on 500 bootstrap resamples; AUC optimism is computed as Apparent minus OOB. Classification performance (sensitivity, specificity, accuracy) is reported at the model-specific operating point maximising Youden's index on OOB predictions. Probabilistic accuracy is summarised using the OOB Brier score and OOB log-loss. Calibration is summarised using the OOB calibration intercept and slope, and expected calibration error (ECE) computed over 10 bins. Pairwise AUC comparisons between models were performed on OOB ROC curves using DeLong's test and are reported as ΔAUC with corresponding p-values.

Table S2. Apples-to-apples OOB classification performance at fixed probability thresholds.

Model	Parameters	Probability threshold				
		0.1	0.2	0.3	0.4	0.5
Clinical model	Sensitivity	1	0.977	0.884	0.837	0.791
	Specificity	0	0.152	0.333	0.455	0.576
	Accuracy	0.566	0.618	0.645	0.671	0.697
	PPV	0.566	0.6	0.633	0.667	0.708
	NPV	NA	0.833	0.688	0.682	0.679
	Youden's index	0	0.129	0.217	0.292	0.367
PET model	Sensitivity	0.977	0.93	0.907	0.907	0.884

	Specificity	0.303	0.515	0.545	0.667	0.667
	Accuracy	0.684	0.75	0.75	0.803	0.789
	PPV	0.646	0.714	0.722	0.78	0.776
	NPV	0.909	0.85	0.818	0.846	0.815
	Youden's index	0.28	0.445	0.452	0.574	0.551
Combined model	Sensitivity	0.977	0.977	0.953	0.884	0.837
	Specificity	0.242	0.394	0.545	0.606	0.667
	Accuracy	0.658	0.724	0.776	0.763	0.763
	PPV	0.627	0.677	0.732	0.745	0.766
	NPV	0.889	0.929	0.9	0.8	0.759
	Youden's index	0.219	0.371	0.498	0.49	0.504

Abbreviations: PPV, positive predictive value; NPV, negative predictive value; OOB, out-of-bag.

This table reports an "apples-to-apples" comparison of the three stepwise logistic regression models for predicting [^{177}Lu]Lu-PSMA treatment eligibility by evaluating each model at the same predefined probability thresholds 0.10–0.50, rather than at model-specific cut-offs. For each threshold, out-of-bag internally validated predictions are dichotomised as eligible if the predicted probability is at least the chosen threshold, and the resulting sensitivity, specificity, accuracy, positive predictive value (PPV), and negative predictive value (NPV) are reported. In addition, Youden's index (J) is provided at each fixed threshold as a summary of the sensitivity-specificity trade-off (J=sensitivity+specificity−1). Reporting fixed-threshold performance facilitates direct clinical comparison because it avoids the interpretability limitations of using different Youden-optimal thresholds for different models.

Table S3. Variable selection stability for the stepwise Clinical, PSMA PET and combined prediction models across bootstrap resamples.

Analysis	Term	Frequency
Analysis 1. Clinical model	**Chaarted_Criteria**	0.93
	**isto**	0.636
	**Age_mPC**	0.564
	**ISUP45vsless**	0.5
	**ARPI_exp_total**	0.426
	**Only_N**	0.374
	**PET_in_mCRPC**	0.316
	**docetaxel_exp_total**	0.316
Analysis 2. Combined model	**SUVmean**	0.824
	**Nles_numb_PET**	0.758
	**ISUP45vsless**	0.738
	**Chaarted_Criteria**	0.616
	**Age_mPC**	0.564
	**Bone_PET**	0.522
	**isto**	0.502
	**N_ln_PET**	0.48
	**docetaxel_exp_total**	0.382
	**PSMA-TV_percentage**	0.38
	**Only_N**	0.33
	**PET_in_mCRPC**	0.244
	**ARPI_exp_total**	0.196
Analysis 3. PET model	**Nles_numb_PET**	0.964

**Bone_PET**	0.838
**SUVmean**	0.808
**PSMA-TV_percentage**	0.294
**N_ln_PET**	0.242

Abbreviations: VIF variance inflation factor; PET positron emission tomography; PSMA prostate-specific membrane antigen; mPC metastatic prostate cancer; mCRPC metastatic castration-resistant prostate cancer; ISUP 4–5 International Society of Urological Pathology grade groups 4–5; ARPI androgen receptor pathway inhibitor; docetaxel_exp_total prior docetaxel exposure; ARPI_exp_total prior ARPI exposure; Chaarted_Criteria CHAARTED high-volume disease criteria; SUVmean mean standardised uptake value; PSMA-TV%_percentage PSMA tumour volume expressed as a percentage; Nles_numb_PET number of PET lesions; Bone_PET presence of bone involvement on PET; N_ln_PET number of PET-positive lymph node lesions; Only_N nodal-only disease; isto histology; PET_in_mCRPC PET performed in the mCRPC setting.

This table summarises variable selection stability for the three stepwise prediction models by reporting, for each candidate predictor, its selection frequency across the internal bootstrap procedure. Selection frequency is defined as the proportion of bootstrap resamples in which a variable was retained in the final model after multicollinearity control (VIF pruning) followed by stepwise selection. Higher frequencies indicate predictors that are more consistently selected and therefore more robust to resampling variability. Results are presented separately for the Clinical, PSMA PET-only, and Combined (Clinical + PSMA PET) models.

Table S4. PSMA volumetric parameters relative to the salivary-gland uptake threshold, by [^{177}Lu]Lu-PSMA eligibility.

Variable	Total (n = 76)	Not candidate (n = 33)	Candidate (n = 43)	p-value*
Salivary gland PSMA-TV (mL, mean ± SD)	40.83 ± 100.59	22.25 ± 105.26	55.08 ± 95.64	0.160
PSMA-TV percentage (mean ± SD)	0.34 ± 0.38	0.18 ± 0.34	0.47 ± 0.36	0.001
Salivary gland PSMA-TV, mean of log ± SD	1.92 ± 1.93	0.73 ± 1.47	2.83 ± 1.74	< 0.001

Abbreviations: PSMA prostate-specific membrane antigen; PSMA-TV PSMA tumour volume; SD standard deviation. *Mann-Whitney U test (Not candidate vs. Candidate).

These volumetric parameters (salivary-gland-thresholded PSMA tumour volume and its percentage of total tPSMA-TV) were moved from Table 1 to this supplementary table for conciseness; values, group comparisons and p-values are unchanged from the original analysis.

Result S1 – Model Development and Performance

S1.1 Clinical Models

Two different multivariable logistic regression models were constructed from 6 clinical variables that were identified from VIFbased collinearity filtering. The first model incorporated all 6 variables (Full model), whereas the second model was constructed by stepwise selection of the 6 variables and consisted of 4 variables – age, volume of disease, ISUP grade group, adenocarcinoma histology (**Table S5**). Both models showed comparable discrimination (AUC 0.820 vs 0.821). In both models, older age or high-volume disease were associated with a higher chance of eligibility, after adjusting for the other variables

(p<0.017) **(Table S5)**. Overall, both models yielded concordant effects, with the stepwise model providing a more parsimonious clinical specification.

S1. Table S5. Models based on clinical variables.

Model	Predictor	OR	95% CI	p-value
Full model (VIF selected), AUC = 0.820	Age at mPC diagnosis	1.11	[1.02 - 1.20]	0.015
	LV disease (CHAARTED criteria)	0.0548	[0.0103 - 0.291]	< 0.001
	Nodal-only disease	3.21	[0.426 - 24.2]	0.258
	ISUP Grade Group 4–5	0.196	[0.0306 - 1.26]	0.085
	Non-neuroendocrine adeno-carcinoma	5.97	[0.736 - 48.4]	0.094
	mHSPC patients at time of PET	2.38	[0.254 - 22.4]	0.448
Stepwise-selected model, AUC = 0.821	Age at mPC diagnosis	1.10	[1.02 - 1.20]	0.017
	LV disease (CHAARTED criteria)	0.0923	[0.0267 - 0.319]	< 0.001
	ISUP Grade Group 4–5	0.210	[0.0359 - 1.23]	0.084
	Non-neuroendocrine adeno-carcinoma	4.61	[0.735 - 28.9]	0.103

S1.2 PET Models

Two PSMA PET–based models were evaluated: a full model incorporating comprehensive PET metrics consisting of 12 variables, and a stepwise-selected model of 4 of those variables (**Table S6**). Both showed high apparent discrimination (AUC 0.906 and 0.895, respectively). In the full PET model, only the lesion burden of 10–20 PSMApositive lesions was significantly associated with higher odds of eligibility after adjusting for the other variables (OR 13.7, p = 0.033) **(Table S6)**. In contrast, 3 of the 4 variables of the stepwise model were associated with eligibility after adjusting for the other variables - Lesion count categories of10–20 lesions (OR 16.9, p = 0.004) or >20 lesions (OR 14.4, p < 0.001), and SUVmean (OR 1.22, p = 0.004). Multicollinearity remained minimal. Overall, both PET-based models consistently identified lesion burden as the dominant determinant of [^{177}Lu]Lu-PSMA eligibility, with SUVmean providing additional discriminative value in the refined stepwise specification.

S2. Table S6. Models based on PSMA-PET parameters.

Model	Predictor	OR	95% CI	p-value
Full model, AUC = 0.906	> 20 PSMA-positive lesions	6.51	[0.722 -58.7]	0.095
	10-20 PSMA-positive lesions	13.7	[1.24 - 152.0]	0.033
	SUVmean	1.26	[0.94 - 1.70]	0.121
	Bone metastases at PSMA-PET	26.7	[0.395 - 1800]	0.127
	Log (Salivary gland PSMA-TV)	1.48	[0.539 - 4.04]	0.449
	> 10 nodes at PSMA-PET	0.49	[0.0481 - 4.99]	0.547
	5-10 nodes at PSMA-PET	0.739	[0.057 - 9.58]	0.817
	No nodal lesions at PSMA-PET	0.622	[0.0885 - 4.37]	0.633
	PSMA-TV percentage	0.43	[0.00610 - 30.3]	0.697
	Log (TLA)	1.16	[0.541 - 2.49]	0.701
	Log(SUVmax of RL)	0.649	[0.0686 - 6.14]	0.706
	Log (Liver SUVmax)	0.772	[0.0690 - 8.63]	0.833
PET stepwise model, AUC = 0.895	> 20 PSMA-positive lesions	14.4	[3.39 - 60.8]	< 0.001
	10-20 PSMA-positive lesions	16.9	[2.47 - 115.0]	0.004

	SUVmean	1.22	[1.07 - 1.39]	0.004
	Bone metastases at PSMA-PET	36.0	[0.70 - 1848.0]	0.075

S1.3 Combined Models

Combined models integrating clinical and PSMA PET parameters showed high apparent discrimination (**Table S7**, full model AUC 0.944; stepwise model AUC 0.935). The full model consisted of 20 variables, and none of these variables were independently associated with eligibility (p>0.1, **Table S7**). The stepwise combined model consisted of 7 variables (**Table S7**), of which two were significantly associated with eligibility after adjusting for the other variables - SUVmean (OR 1.22, p = 0.015), and prior docetaxel exposure (OR 0.100, p = 0.028). Although both models have similar AUC, the full model is likely to have some degree of overfitting, given the relatively small sample size, the large number of candidate predictors, the wide confidence intervals and non-significant individual predictors. **These are apparent, in-sample values and are optimistic; the internally validated out-of-bag AUCs were 0.731, 0.817 and 0.829 for the clinical, PSMA PET and combined models respectively, and no pairwise difference reached statistical significance.**

S3. Table S7. Models combining clinical variables and PSMA-PET parameters:.

Model	Predictor	OR	95% CI	p-value
Full model, AUC =0.944	Age at mPC diagnosis	1.06	[0.894 - 1.25]	0.510
	LV disease (CHAARTED criteria)	0.108	[0.00616 - 1.90]	0.129
	Nodal-only disease	2.45	[0.0458 - 131.0]	0.659
	ISUP Grade Group 4–5	0.0622	[0.00213 - 1.82]	0.107
	Non-neuroendocrine adenocarcinoma	26.0	[0.36 - 1883.0]	0.136
	mHSPC patients at time of PET	0.554	[0.00841- 36.5]	0.782
	Prior ARPI exposure	1.12	[0.0178 - 70.5]	0.957
	Prior docetaxel exposure	0.0636	[0.000862 - 4.69]	0.209
	SUVmean	1.48	[0.88 - 2.50]	0.138
	PSMA-TV percentage	0.0664	[0.000138 - 32.0]	0.389
	> 20 PSMA-positive lesions	14.9	[0.542 - 407]	0.110
	10-20 PSMA-positive lesions	22.5	[0.181 - 2793]	0.206
	> 10 nodes at PSMA-PET	0.225	[0.00738 - 6.87]	0.393
	5-10 nodes at PSMA-PET	0.789	[0.0139 - 44.7]	0.908
	No nodal lesions at PSMA-PET	1.30	[0.0819 - 20.6]	0.853
	Bone metastases at PSMA-PET	114.0	[0.425 - 30791.0]	0.097
	Log (SUVmax of RL)	0.693	[0.00590 - 81.4]	0.880
	Log (TLA)	0.807	[0.232 - 2.81]	0.736
	Log (Salivary gland PSMA-TV)	2.03	[0.515 - 7.96]	0.312
	Log (Liver SUVmax)	0.108	[0.00128 - 9.15]	0.326
Combined stepwise model, AUC =0.935	LV disease (CHAARTED criteria)	0.199	[0.0245 - 1.61]	0.130
	Prior docetaxel exposure	0.10	[0.0129 - 0.776]	0.028
	SUVmean	1.22	[1.04 - 1.42]	0.015
	> 20 PSMA-positive lesions	7.78	[0.799 - 75.8]	0.077
	10-20 PSMA-positive lesions	9.91	[0.912 - 108.0]	0.060
	Bone metastases at PSMA-PET	19.6	[0.374 - 1023.0]	0.141
	Log (TLA)	1.77	[0.957 - 3.29]	0.069

Result S2 - Internal Validation, Classification Profiles, and Model Comparison

Internal validation was performed using 500 bootstrap resamples with performance estimated from resampling-based out-of-bag (OOB) predictions, ensuring that each patient's predicted probability was generated only from models that did not include that patient in the corresponding bootstrap sample. Under this framework, discrimination increased substantially when moving from the clinical-only model to PET-based specifications, with OOB AUCs of 0.731 (clinical), 0.817 (PSMA PET), and 0.829 (combined). This pattern indicates that the main discriminative signal is captured by PET-derived predictors, with only a modest incremental contribution from clinical variables once PET information is available.

Classification behaviour at the model-specific OOB Youden thresholds showed distinct operating points. The clinical model yielded a more conservative profile, with specificity (0.818) exceeding sensitivity (0.628) and an overall accuracy of 0.711. The PSMA PET model provided the highest sensitivity (0.837) with specificity 0.758 and accuracy 0.803, consistent with improved identification of eligible patients and a more balanced trade-off between false negatives and false positives. The combined model shifted toward higher specificity (0.818) with sensitivity 0.767 and accuracy 0.789, reflecting a modest rebalancing relative to PET alone rather than a large net change in classification accuracy. In absolute terms, PET-based models improved accuracy by approximately 8–9 percentage points compared with the clinical model (0.803 and 0.789 vs 0.711).

Probabilistic accuracy metrics supported the same ordering. The Brier score decreased from 0.2085 (clinical) to 0.1636 (PSMA PET) and 0.1640 (combined), indicating improved overall probability accuracy for PET-based models, with near-identical values for PET and combined. Log-loss was lowest for the combined model (0.5135) compared with PSMA PET (0.5281) and clinical (0.6138), suggesting slightly improved probabilistic sharpness for the combined model despite minimal separation from PET alone.

For statistical comparison of discrimination, DeLong's test was applied to paired ROC curves derived from OOB predictions. Differences were small and not statistically significant for combined vs PSMA PET (ΔAUC 0.012, $p = 0.624$) and for PSMA PET vs clinical (ΔAUC 0.087, $p = 0.203$), while combined vs clinical showed borderline evidence of improvement (ΔAUC 0.099, $p = 0.081$), consistent with limited sample size and uncertainty around AUC estimates. Overall, internal validation supports a robust practical improvement from clinical modelling to PET-based modelling, while the incremental gain from adding clinical variables on top of PET is best characterised as modest. Graphical comparisons of OOB ROC curves and calibration are provided in Figure 5 and Figures S3–S4.

Result S3 - Sensitivity analyses for the prior-docetaxel coefficient

Because the association between prior docetaxel exposure and PSMA PET-defined eligibility rested on 25 exposed patients and carried a wide confidence interval, two sensitivity analyses were performed. First, the combined model was refitted using Firth's penalised likelihood, which reduces small-sample bias. Second, the combined model was refitted after removing prior docetaxel exposure altogether. Formal separation was not present: no cell of the docetaxel-by-eligibility table was empty and the eligibility rate was virtually identical in exposed and unexposed patients, so the multivariable coefficient reflects sparse-data bias and adjustment for PSMA PET burden rather than quasi-complete separation.

Table S8. Firth penalised logistic regression of the combined stepwise model.

Predictor	Firth OR	95% CI	p-value
Chaarted_Criterialow	0.235	0.0334 to 1.47	0.120
docetaxel_exp_total1	0.156	0.02147 to 0.8348	0.029
SUVmean	1.159	1.037 to 1.361	0.007
Nles_numb_PET>20	5.043	0.7425 to 43.68	0.099
Nles_numb_PET10_20	5.383	0.7816 to 49.55	0.088
Bone_PET1	6.712	0.5553 to 408.9	0.144
log_TLA	1.614	0.9276 to 2.82	0.089

Result S4 - Disease-state subgroups (mHSPC versus mCRPC)

Because the cohort combined patients imaged in the mHSPC and mCRPC settings, eligibility rates and model performance are reported descriptively within each subgroup. The models were not refitted within subgroups: with approximately 20 events per subgroup this would leave about three events per variable, which would not support a credible model. These subgroup estimates are descriptive only and carry wide uncertainty.

S6. Table S9. Descriptive eligibility rates and internally validated discrimination within the mHSPC and mCRPC subgroups.

Model	Subgroup	n	Eligible	Eligible (%)	OOB AUC
Clinical	mHSPC	40	20	50	0.835
Clinical	mCRPC	36	23	63.9	0.562
PET	mHSPC	40	20	50	0.865
PET	mCRPC	36	23	63.9	0.739
Combined	mHSPC	40	20	50	0.912
Combined	mCRPC	36	23	63.9	0.753

Result S5 - CHAARTED volume adjusted for PSMA-positive lesion count

To test whether the association between CHAARTED low-volume disease and lower eligibility is largely a restatement of a lower PSMA-positive lesion count, the two variables were modelled together. The CHAARTED association was substantially attenuated after adjustment and no longer reached significance, indicating that much of the apparent effect of conventional-imaging disease volume is explained by PSMA-positive lesion burden rather than acting independently of it.

Table S10. Low-volume disease before and after adjustment for the number of PSMA-positive lesions.

Model	OR for low-volume disease	95% CI	p-value
CHAARTED alone	0.119	0.03958 to 0.3607	0.0002
CHAARTED + PSMA-positive lesion count	0.450	0.1063 to 1.905	0.2782

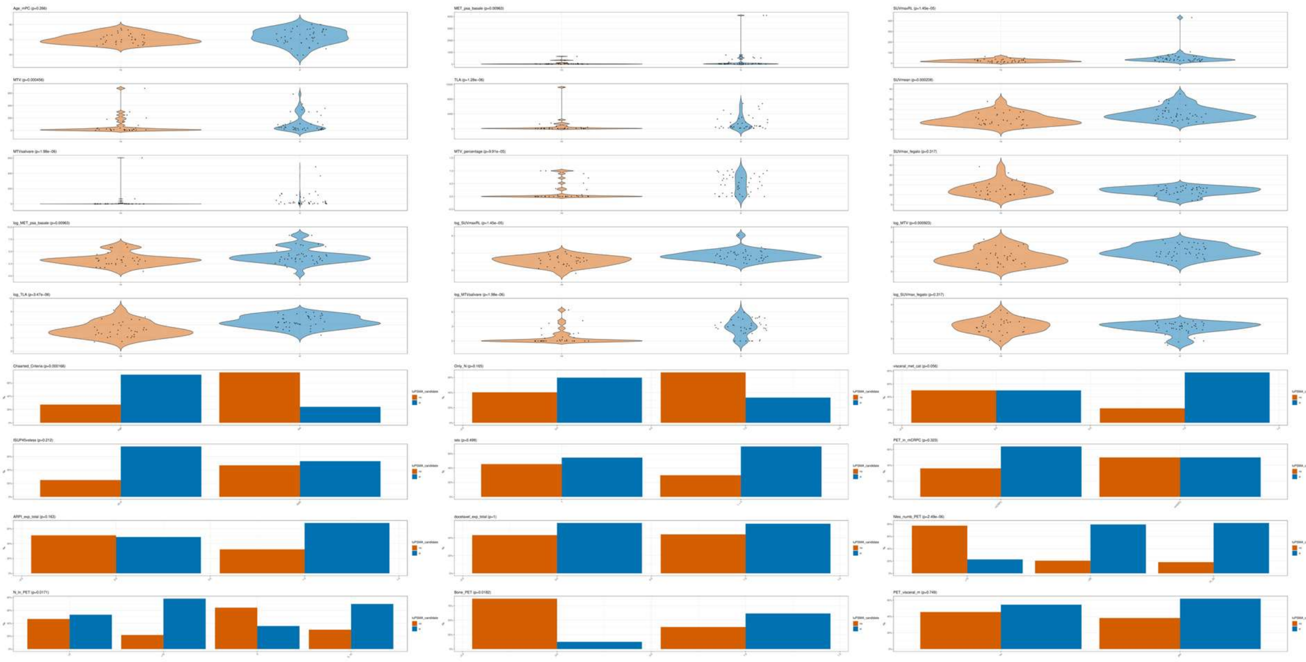

Figure S1. Distribution of key clinical, pathological, and PSMA PET parameters according to [177Lu]Lu PSMA eligibility. PCa, prostate cancer; mPC, metastatic prostate cancer; PSA, prostate-specific antigen; PSMA, prostate-specific membrane antigen; PET, positron emission tomography; SUV, standardised uptake value; SUVmaxRL, maximum SUV of the reference lesion; SUVmean, mean SUV; tPSMA-TV, total PSMA tumour volume; TLA, total lesion activity; RLT, radioligand therapy.

Violin plots and bar plots illustrating the distribution of selected clinical and PSMA PET–derived variables in patients deemed eligible versus ineligible for [177Lu]Lu PSMA RLT. Panels display: age at metastatic prostate cancer diagnosis (Age_mPC), baseline PSA at metastatic presentation (MET_psa_basale), maximum standardised uptake value of the reference lesion (SUVmaxRL), total PSMA tumour volume (tPSMA-TV), total lesion activity (TLA), and mean standardised uptake value (SUVmean). p values from univariable comparisons between eligible and ineligible groups are reported for each parameter

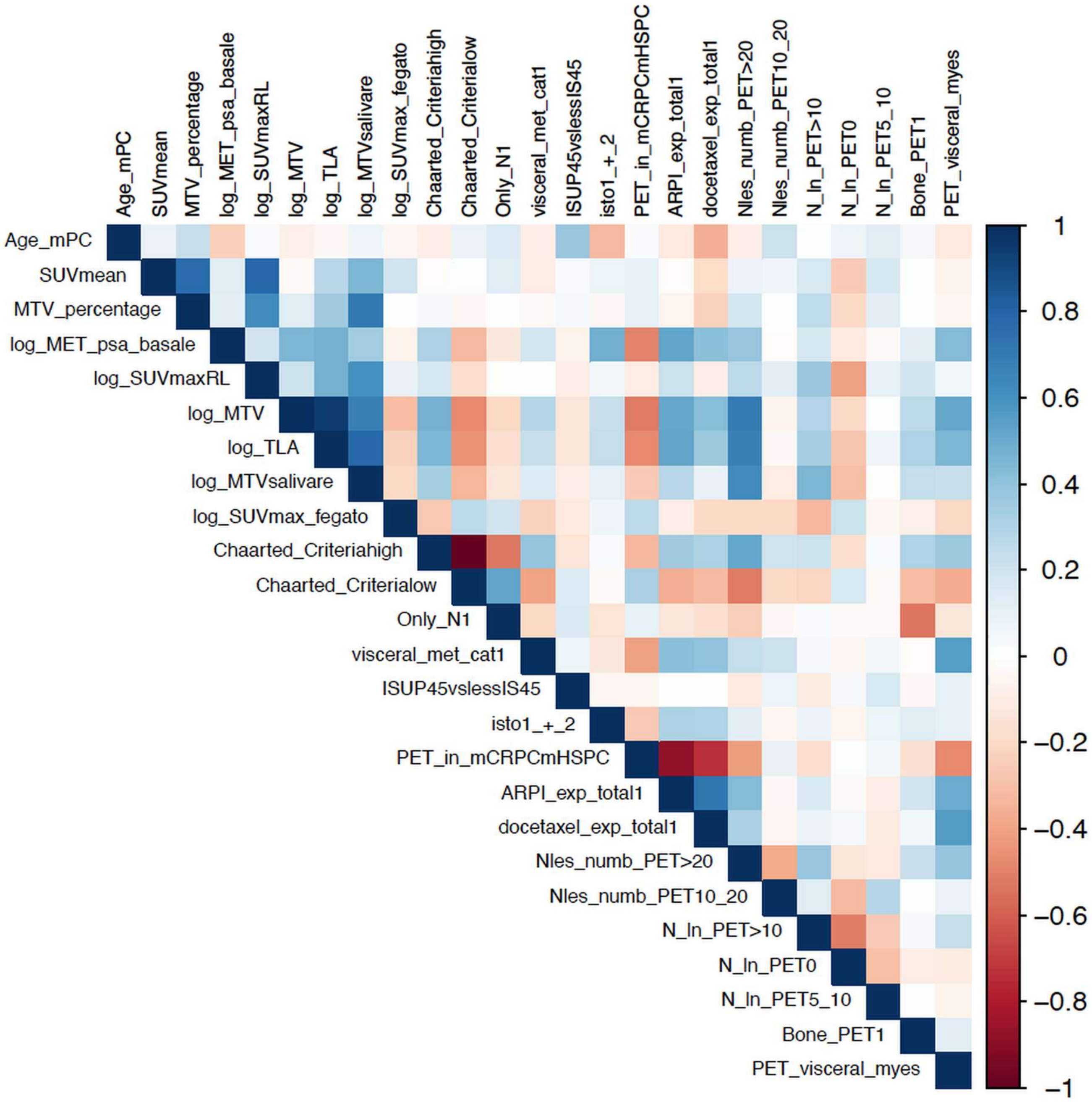

Figure S2. Correlation matrix of clinical, pathological, and PSMA PET parameters. Age_mPC, age at diagnosis of metastatic prostate cancer; ARPI, androgen receptor pathway inhibitor; ARPI_exp_total1, prior ARPI exposure (yes vs no); CHAARTED_Criterialow / CHAARTED_Criteriahigh, low- / high-volume disease according to CHAARTED criteria; docetaxel_exp_total1, prior docetaxel exposure (yes vs no); ISUP45vslessIS45, ISUP grade ≥4 vs ≤3; log_MET_psa_basale, log-transformed baseline PSA at metastatic presentation; log_tPSMA-TV, log-transformed total PSMA tumour volume; log_PSMA-TV salivare, log-transformed PSMA-TVsal; log_SUVmax_fegato, log-transformed hepatic SUVmax; log_SUVmaxRL, log-transformed maximum SUV of the reference lesion; log_TLA, log-transformed total lesion activity; tPSMA-TV, total PSMA tumour volume; PSMA-TV%_percentage, tPSMA-TV expressed as a percentage of total tumour burden; Nles_numb_PET10_20, 10–20 PSMApositive lesions on PET; Nles_numb_PET>20, >20 PSMApositive lesions on PET; N_ln_PET0, no PSMApositive lymph nodes; N_ln_PET5_10, 5–10 PSMApositive lymph nodes; N_ln_PET>10, >10 PSMApositive lymph nodes; Only_N1, nodal-only metastatic disease at baseline; PET_in_mCRPCmHSPC, timing of PSMA PET (mCRPC vs mHSPC); PET_visceral_myes, visceral metastases on PSMA PET (yes vs no); PSA, prostate-specific antigen; PSMA,

prostate-specific membrane antigen; SUV, standardised uptake value; SUVmaxRL, maximum SUV of the reference lesion; SUVmean, mean SUV; TLA, total lesion activity.

Heatmap of pairwise correlation coefficients between clinical, pathological, demographic, and PSMA PET–derived variables in the overall cohort. Colour intensity indicates the strength and direction of the correlation, with stronger positive and negative correlations shown by more saturated colours. This analysis was used to identify highly intercorrelated variables (r > 0.7), which were subsequently excluded from multivariable models to reduce redundancy and support robust variable selection.

The correlation heat-map revealed distinct patterns of association among clinical and PSMA PET-derived variables using one-hot encoding for categorical predictors. Volumetric tumour burden metrics demonstrated strong intercorrelations, forming a cohesive high collinearity cluster with tPSMA-TV and TLA showing high correlation (r ≈ 0.86), reflecting their biological relationship as TLA represents the product of tPSMA-TV and SUVmean. PSMA-TV percentage similarly correlated strongly with both tPSMA-TV (r ≈ 0.78) and TLA (r ≈ 0.70), as expected given its derivation as normalised tPSMA-TV. This cluster extended to include log_PSMA-TVsal, log_SUVmaxRL, SUVmean, and the "Nles_numb_PET > 20" categorical dummy (correlations r = 0.6-0.9), all describing the same biological construct of total PSMA tumour burden. The categorical encoding provided additional granular insights, with the "Nles_numb_PET > 20" dummy variable correlating strongly (~0.6) with volumetric metrics while maintaining independence from the "10-20" category, indicating that extreme lesion counts capture unique information beyond simple volume measurements. Modest correlations (r ≈ 0.3) were observed between bone and visceral metastases dummies and the tPSMA-TV cluster, suggesting that disease location partially overlaps with tumour burden but retains independent prognostic value.

In contrast, uptake intensity measures including SUVmax of reference lesions and liver SUVmax showed modest intercorrelations (r = 0.25-0.40) but minimal association with volumetric parameters (r ≤ 0.20), indicating they capture distinct biological characteristics related to intrinsic aggressiveness rather than disease bulk. Clinical variables including age, ISUP grade, prior therapies, scan timing, and baseline PSA demonstrated minimal correlations with imaging features and each other (|r| < 0.3), suggesting these demographic and biochemical factors provide orthogonal information independent of PSMA PET-derived metrics and can be safely included in multivariable models without collinearity concerns

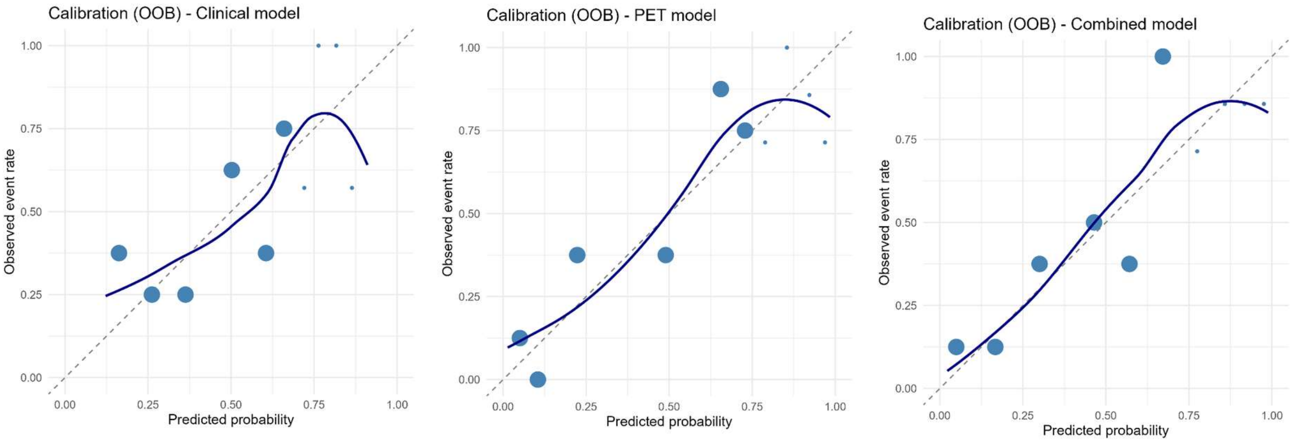

Figure S3. Calibration plots for the three stepwise multivariable models predicting [177Lu]Lu-PSMA treatment eligibility using internally validated outofbag (OOB) predictions. OOB out-of-bag; PET positron emission tomography; PSMA prostate-specific membrane antigen; [177Lu]Lu-PSMA lutetium177–labelled PSMA-targeted radioligand.

Predicted probabilities (xaxis) are grouped into probability strata and plotted against the corresponding observed event proportions (yaxis). The dashed grey diagonal represents perfect calibration (observed = predicted). Blue bubbles represent the observed proportion within each stratum, with bubble size proportional to the number of patients in that bin, while the solid blue line shows a smoothed calibration curve fitted to the OOB predictions. Overall calibration is acceptable but not perfect: the clinical model shows more irregularity and larger departures from the ideal line, especially at low-to-intermediate predicted risks, whereas PSMA PET-only and combined models track the diagonal more closely across most of the probability range, with only mild miscalibration and a tendency toward slight over-prediction at higher estimated risks.

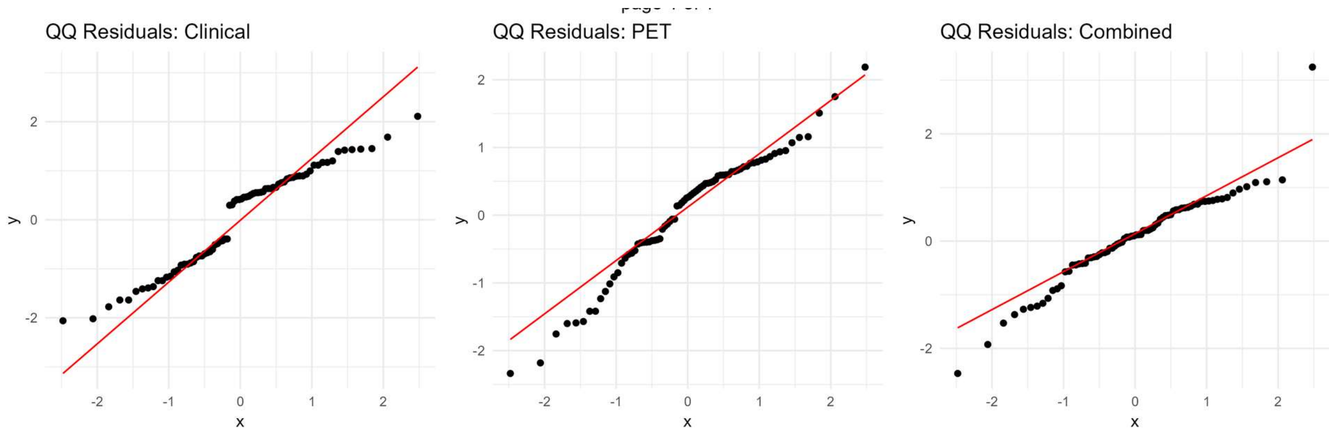

Figure S4. Quantile–quantile plots of residuals for stepwise clinical, PSMA PET, and combined models. QQ, quantile–quantile PET, positron emission tomography PSMA, prostatespecific membrane antigen [^{177}Lu]Lu-PSMA, lutetium177–labelled PSMAtargeted radioligand.

Quantile–quantile (QQ) plots of model residuals for the three stepwise multivariable models predicting [^{177}Lu]Lu-PSMA treatment eligibility. Black points represent the empirical quantiles of the residuals, and the red line denotes the theoretical reference distribution. The clinical and combined models show residuals that lie close to the reference line over most of the distribution, indicating no major deviations from model assumptions, with only minor departures in the tails. The PSMA PET model displays slightly larger deviations in the lower tail, suggesting heavier tails or mild asymmetry, but residual behaviour remains acceptable overall.

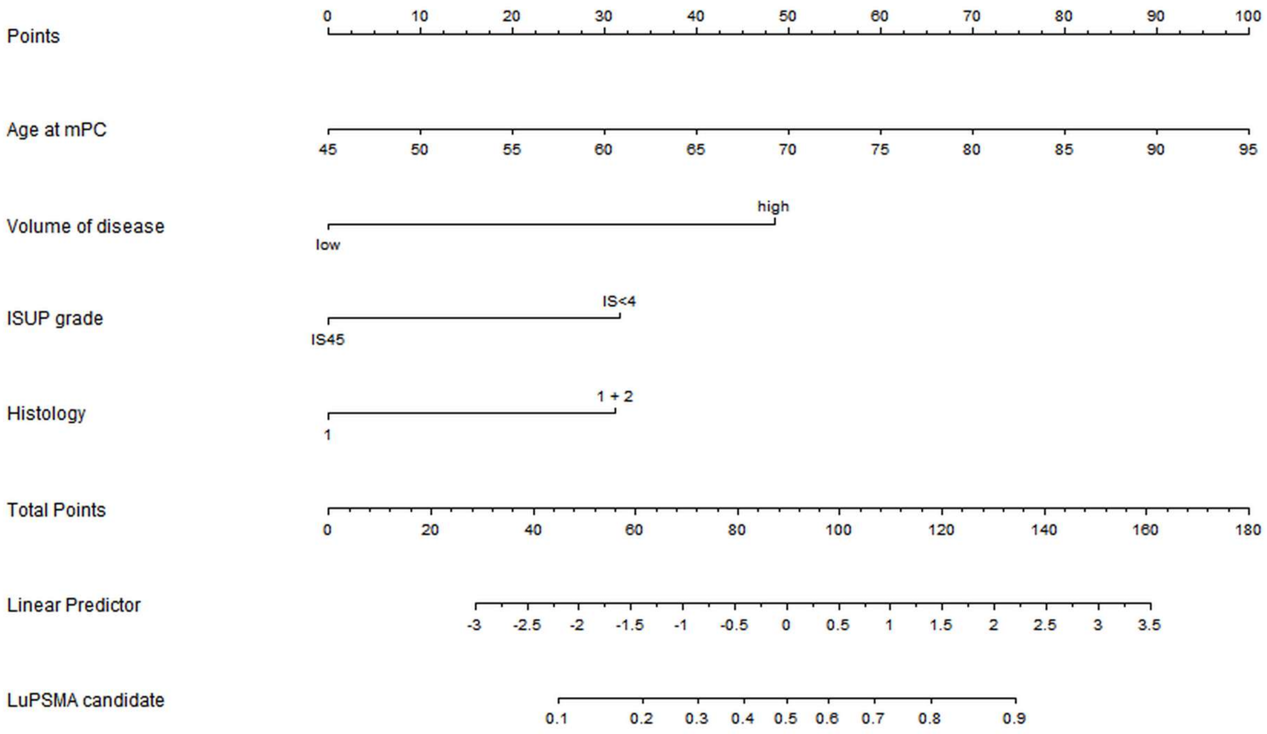

Figure S5. Nomogram of the clinical-only stepwise model estimating the probability of eligibility for [^{177}Lu]Lu PSMA therapy in de novo metastatic prostate cancer.

Nomogram of the clinical-only stepwise logistic regression model estimating the probability of eligibility for [^{177}Lu]Lu-PSMA radioligand therapy in patients with de novo metastatic prostate cancer. The nomogram integrates clinical variables retained in the final model, including age at metastatic prostate cancer diagnosis, disease volume according to CHAARTED criteria, ISUP grade group, and histological subtype. For each variable, the corresponding number of points is assigned on the upper scale; the sum of points is then projected onto the total points axis and translated into an individualised probability of [^{177}Lu]Lu-PSMA eligibility on the lower scale.

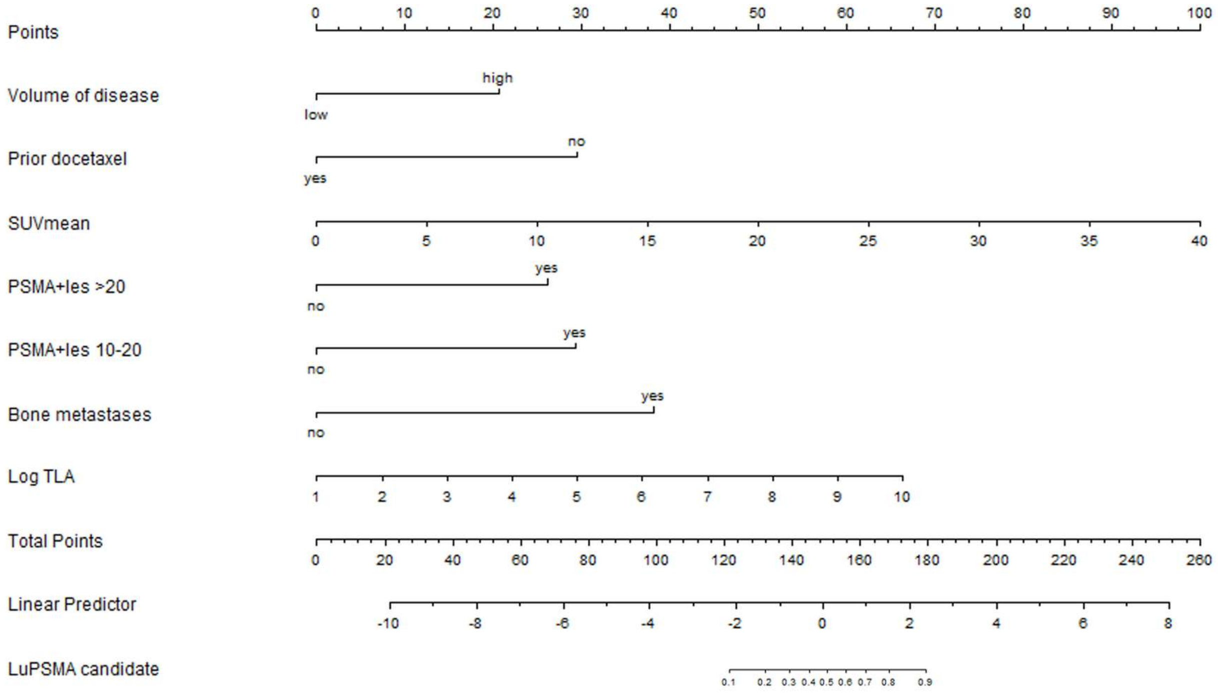

Figure S6. Nomogram of the combined stepwise model estimating the probability of eligibility for [^{177}Lu]Lu-PSMA therapy in de novo metastatic prostate cancer.

The nomogram integrates clinical and PSMA PET-derived variables retained in the final multivariable model, including disease volume, prior docetaxel exposure, SUVmean, PSMA lesion burden, bone metastases, and log TLA. Points assigned to each variable are summed and mapped to the total points scale to derive an individualised probability of [^{177}Lu]Lu-PSMA eligibility.

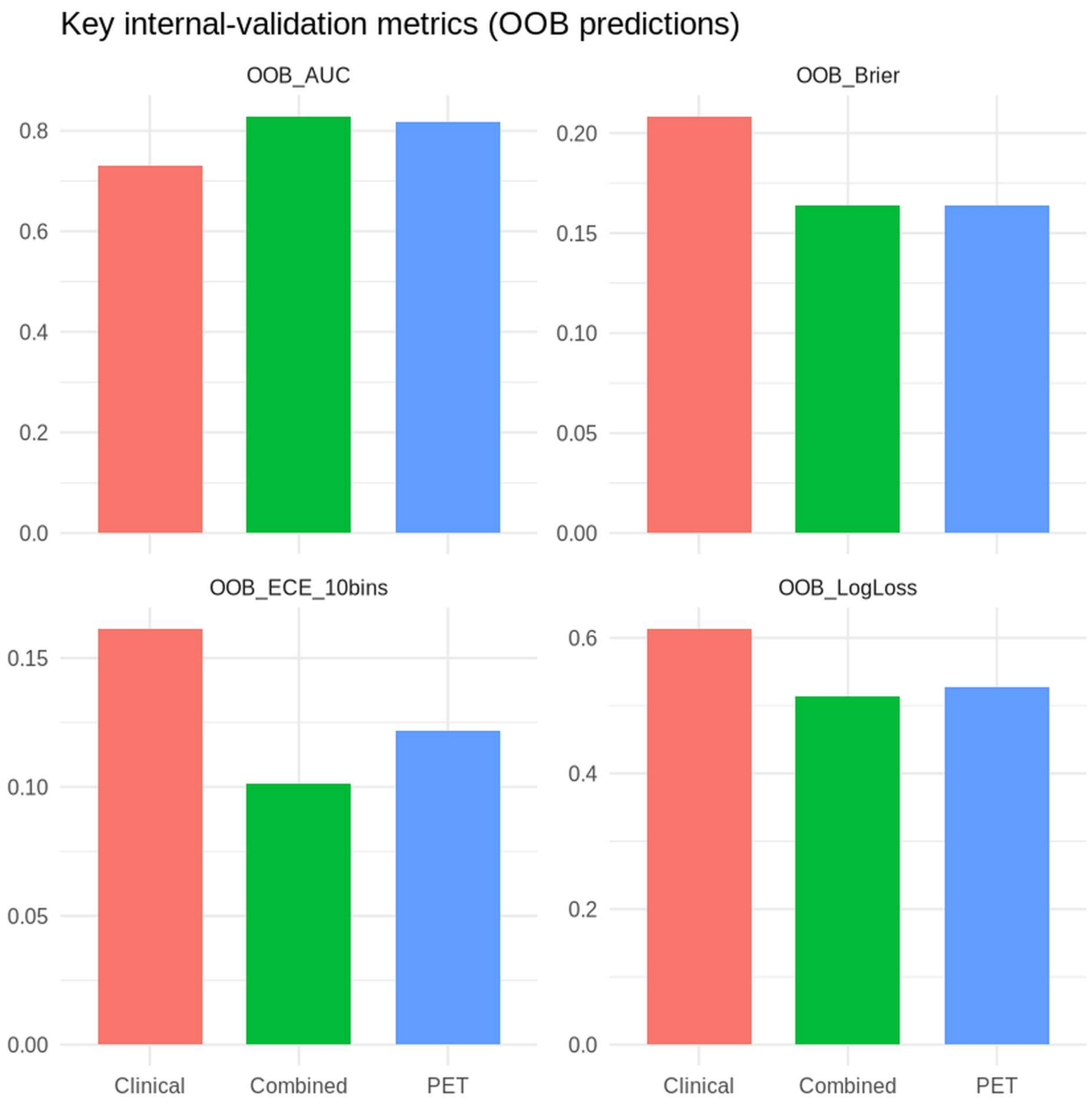

Figure S7. Internally validated OOB performance summary across stepwise models. AUC, area under the receiver-operating characteristic curve; ECE, expected calibration error; LogLoss, logarithmic loss; OOB, out-of-bag; PET, positron emission tomography; PSMA, prostate-specific membrane antigen; [^{177}Lu]Lu-PSMA, lutetium177–labelled PSMA-targeted radioligand.

Bar plots summarising key internal bootstrap validation results based on out-of-bag (OOB) predictions for the three stepwise logistic regression models predicting eligibility for [^{177}Lu]Lu-PSMA therapy. Each panel reports a different OOB metric, enabling direct comparison of discrimination, overall probabilistic error, calibration error, and proper scoring performance across the Clinical (pink), PSMA PET-only (blue), and Combined clinical + PSMA PET (green) models.

In the OOB AUC panel, discrimination is lowest for the clinical-only model (moderate performance) and markedly higher for both PET-based models, with the combined model showing only a small AUC advantage over PET-only. In the OOB Brier score panel (lower is better), PSMA PET-only and combined are essentially overlapping and clearly

better than the clinical model, indicating that PET-based specifications substantially reduce overall probability error, while adding clinical predictors to PET does not materially change the Brier score. In the OOB ECE (10 bins) panel (lower is better), the combined model has the lowest calibration error, followed by PET-only, with the clinical model showing the highest ECE, consistent with slightly more stable probability calibration when clinical variables are added to PET. Finally, the OOB log-loss panel (lower is better) again favours PET-based models; the combined model is marginally best, with PET-only close behind and the clinical model worst, suggesting a modest improvement in probabilistic sharpness for the combined specification.

Overall, this figure visually supports the main validation message: moving from clinical-only to PET-based modelling yields the major performance gain, whereas the combined model provides only incremental improvements over PET-only, most evident in calibration error and log-loss rather than in discrimination or Brier score.

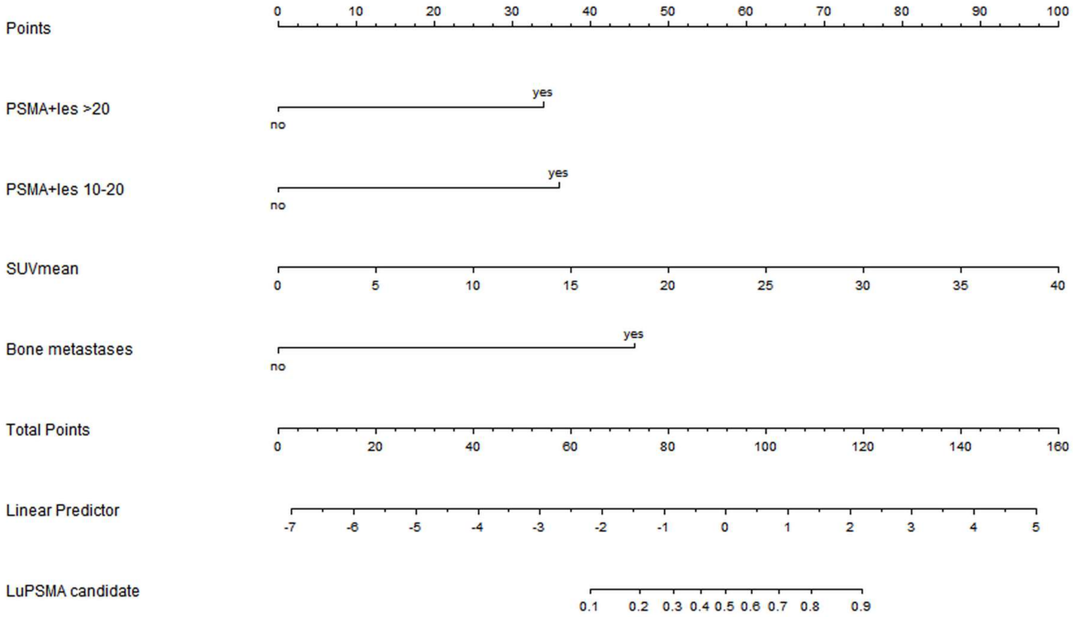

Figure S8. Nomogram of the PSMA PET stepwise model estimating the probability of eligibility for [^{177}Lu]Lu-PSMA therapy in de novo metastatic prostate cancer.
